# Biochemical and structural explorations of α-hydroxyacid oxidases reveal a four-electron oxidative decarboxylation reaction

**DOI:** 10.1107/S2059798319009574

**Published:** 2019-07-30

**Authors:** Hsien-Wei Yeh, Kuan-Hung Lin, Syue-Yi Lyu, Yi-Shan Li, Chun-Man Huang, Yung-Lin Wang, Hao-Wei Shih, Ning-Shian Hsu, Chang-Jer Wu, Tsung-Lin Li

**Affiliations:** a Genomics Research Center, Academia Sinica, Taipei 115, Taiwan; b Institute of Biochemistry and Molecular Biology, National Yang-Ming University, Taipei 112, Taiwan; c Department of Food Science, National Taiwan Ocean University, Keelung 202, Taiwan; d Biotechnology Center, National Chung Hsing University, Taichung City 402, Taiwan

**Keywords:** mandelate oxidase, flavin mononucleotide, oxidative decarboxylation, hydride transfer, α-hydroxyacids

## Abstract

*p*-Hydroxymandelate oxidase (Hmo) is a flavin mononucleotide (FMN)-dependent enzyme that oxidizes mandelate to benzoylformate. How the FMN-dependent oxidation is executed by Hmo remains unclear at the molecular level. A continuum of snapshots from crystal structures of Hmo and its mutants in complex with physiological/nonphysiological substrates, products and inhibitors provides a rationale for its substrate enantioselectivity/promiscuity, its active-site geometry/reactivity and its direct hydride-transfer mechanism. A single mutant, Y128F, that extends the two-electron oxidation reaction to a four-electron oxidative decarboxylation reaction was unexpectedly observed. Biochemical and structural approaches, including biochemistry, kinetics, stable isotope labeling and X-ray crystallo­graphy, were exploited to reach these conclusions and provide additional insights.

## Abstract 2

Structural and enzymological explorations of α-hydroxyacid oxidases uncover new flavin mononucleotide-mediated reactions and intermediates.

## 1. Introduction   

**Figure 1:**
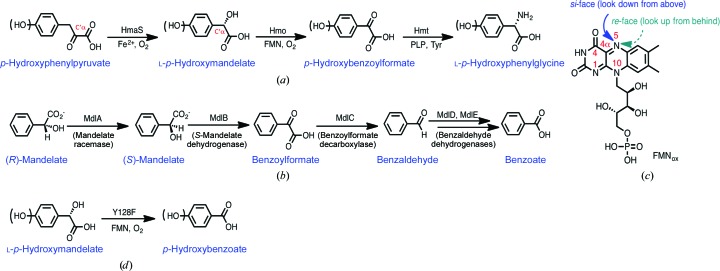
Pathways and structures. (*a*) Anabolic pathway of l-*p*-hydroxyphenylglycine. (*b*) Metabolic pathway of (*R*)-mandelate; MdlB is also referred to as *S*-­mandelate dehydrogenase (MDH). (*c*) Chemical structure of oxidized flavin mononucleotide (FMN_ox_). (*d*) The direct conversion of mandelate to benzoate by the Y128F mutant via a four-electron oxidative decarboxylation reaction. The numbering system is shown in red for FMN and phenylpyruvate; the *si*-face and *re*-face prochirality relative to the N5 atom of the isoalloxazine ring is indicated in blue and green, respectively.

l-*p*-Hydroxyphenylglycine (l-*p*-HPG), a nonproteinogenic α-amino acid, exists in many clinically important glycopeptide natural products, for example vancomycin (van Wageningen *et al.*, 1998 ▸), teicoplanin (Li *et al.*, 2004 ▸) and ramoplanin (McCafferty *et al.*, 2002 ▸; Walker *et al.*, 2005 ▸). The peptide cores of these glycopeptides recruit two, three or six l-*p*-HPG units, respectively, as the site for glycosylation in addition to their structural roles (Supplementary Fig. S1). The biosynthesis of l-*p*-HPG requires three enzymes, (*S*)-*p*-hydroxymandelate synthase (HmaS), (*S*)-*p*-hydroxymandelate oxidase (Hmo) and l-*p*-hydroxyphenylglycine transaminase (HpgT): HmaS transforms phenylpyruvate to (*S*)-mandelate, Hmo catalyzes the conversion of (*S*)-mandelate to benzoylformate and HpgT then transaminates benzoylformate to l-*p*-HPG [Fig. 1 ▸(*a*)] (Al Toma *et al.*, 2015 ▸; Hubbard *et al.*, 2000 ▸; Li *et al.*, 2001 ▸). In the mandelate metabolism, (*R*)-mandelate is converted to (*S*)-mandelate by mandelate racemase, and (*S*)-mandelate is then oxidized to benzoylformate by (*S*)-mandelate dehydrogenase (MDH/MdlB). Benzoylformate subsequently undergoes decarboxylation to benzaldehyde by a thiamine-pyrophos­phate (ThDP)-dependent decarboxylase. Benzaldehyde is further oxidized by dehydrogenases to benzoate [Fig. 1 ▸(*b*)] (Petsko *et al.*, 1993 ▸; Tsou *et al.*, 1990 ▸).

Hmo shares high sequence homology with flavin mononucleotide (FMN)-dependent oxidoreductases (Supplementary Fig. S2) such as MDH (Lehoux & Mitra, 1999 ▸), l-lactate oxidase (LO; Lockridge *et al.*, 1972 ▸), flavocytochrome *b*_2_ (FCB2; Xia & Mathews, 1990 ▸), glycolate oxidase (GO; Lindqvist, 1989 ▸), l-lactate monooxygenase (LMO; Giegel *et al.*, 1990 ▸; Maeda-Yorita *et al.*, 1995 ▸), long-chain l-α-hydroxy­acid oxidase (LCHAO; Cunane *et al.*, 2005 ▸), human glycolate oxidase (hGOX; Murray *et al.*, 2008 ▸) and human l-α-hydroxyacid oxidase 2 (HAOX2; Jones *et al.*, 2000 ▸). With the exception of LMO, all of these oxidases execute two-electron oxidation of α-hydroxyacids to the corresponding α-ketoacids following the classic ping-pong reaction mechanism. In contrast to β-ketoacids, α-ketoacids are devoid of a suitably placed electron sink to stabilize the negative charge developed upon decarboxylation. For the decarboxylation of α-ketoacids, a common solution in nature is to utilize the cofactor ThDP or pyridoxal phosphate (PLP), as exemplified in the central metabolism (Perham *et al.*, 2002 ▸). To the best of our knowledge, LMO is the only ThDP/PLP/NADPH-independent enzyme that performs oxidative decarboxylation of l-lactate en route from pyruvate to acetate at the expense of one molecule of O_2_, with the concomitant production of CO_2_ and H_2_O, and thus is referred to as a monooxygenase (Ghisla & Massey, 1989 ▸). When lactate is oxidized to pyruvate, the oxidized FMN [FMN_ox_; Fig. 1 ▸(*c*)] is concertedly reduced to FMN_red_ upon receiving a pair of electrons: the reductive half-reaction. FMN_red_ is instantly oxidized to FMN_ox_ by O_2_ to form hydrogen peroxide (H_2_O_2_): the oxidative half-reaction (Fagan & Palfey, 2010 ▸; Romero *et al.*, 2018 ▸). Given that the dissociation of pyruvate from LMO is a slow step, the H_2_O_2_ generated at the active site acts on pyruvate to form acetate via oxidative decarboxylation (Giegel *et al.*, 1990 ▸; Lopalco *et al.*, 2016 ▸). Aside from this non-ping-pong kinetic description, how H_2_O_2_ mediates the oxidative decarboxylation of α-ketoacids remains elusive (Choong & Massey, 1980 ▸; Ghisla & Massey, 1977 ▸; Lockridge *et al.*, 1972 ▸; Walsh *et al.*, 1973 ▸). In this study, we explored Hmo and its mutants to gain insights into the reaction center [Fig. 1 ▸(*d*); see below]. A biochemical/structural biology approach renders various liganded structures of the proteins, which in combination deconvolve reactive/unstable Hmo and its mutant-mediated reactions at the molecular level.

## 2. Materials and methods   

### 2.1. Protein expression and purification   

The ligated plasmids of native *hmo* and its mutants were transformed into *Escherichia coli* BL21(DE3) cells and cultured in 1 l LB medium containing 50 mg l^−1^ kanamycin at 37°C until the *A*_600_ reached 0.6. For protein expression and purification, 200 µl of 1.0 *M* isopropyl β-d-thiogalacto­pyranoside (IPTG) was added to the cultured *E. coli* medium for a further 12 h at 16°C. The cells were harvested by centrifugation, resuspended in 40 ml binding buffer (20 m*M* HEPES pH 7.5, 500 m*M* NaCl, 10 m*M* imidazole, 10% glycerol) and ruptured by sonication. Cell debris was spun down by centrifugation (20 000 rev min^−1^, 30 min, 4°C) and the supernatants were applied onto an Ni^2+^–NTA resin column for further purification. The column was washed twice with buffers at different concentrations (first wash, 20 m*M* HEPES pH 8.0, 500 m*M* NaCl, 40 m*M* imidazole; second wash, 20 m*M* HEPES pH 8.0, 500 m*M* NaCl, 80 m*M* imidazole) before eluting the target protein with 15 ml elution buffer (20 m*M* HEPES pH 8, 500 m*M* NaCl, 250 m*M* imidazole). All proteins were further purified by size-exclusion chromatography on an ÄKTA FPLC system equipped with a HiLoad 16/60 Superdex 200 column under an isocratic condition (20 m*M* HEPES pH 8.0, 100 m*M* NaCl). Protein purity was examined by SDS–PAGE. Protein concentrations were estimated by the Bradford assay using BSA as a standard.

### 2.2. Crystallization and data collection   

Hmo and its Y128F mutant were crystallized using the hanging-drop vapor-diffusion method at 20°C. Each protein was concentrated to 7 mg ml^−1^ in 50 m*M* HEPES pH 8.0 buffer solution and was then mixed with the same volume of reservoir solution (35% Tascimate, 0.1 *M* bis-Tris propane pH 7.0). Crystals generally formed after a five-day incubation. Before X-ray diffraction data collection, the crystals were transferred to the same mother liquor containing 20% glycerol as a cryoprotectant. For Hmo or its Y128F mutant in complex with 4-hydroxy-(*S*)-mandelate (4HSMA), (*S*)-mandelate (SMA), benzoylformate (BF), benzoic acid (BA), benzaldehyde (BZ), (*S*)-propionic acid (SPPA), l-4-hydroxyphenylglycine (LPG) and phenylpyruvate, X-ray data sets were recorded after soaking protein crystals with 10–30 m*M* of the given substrate for 10 min to 24 h in the reservoir solution. All diffraction data were collected using ADSC Quantum 315 or MX300-HE CCD detectors at an operating temperature of 100 K on beamlines 13B1, 13C1, 15A1 and 05A at the National Synchrotron Radiation Research Center (NSRRC), Taiwan or on beamline 44XU at SPring-8, Japan. Data were indexed, and scaled with the *HKL*-2000 package (Otwinowski & Minor, 1997 ▸). The Y128F mutant crystals belonged to space group* I*422, with unit-cell parameters *a* = 137.565, *b* = 137.565, *c* = 112.087 Å.

### 2.3. Structure determination and refinement   

The crystal phase information for Hmo was obtained by molecular replacement using *Phaser-MR* from the *CCP*4 suite (McCoy *et al.*, 2007 ▸; Winn *et al.*, 2011 ▸). Hydroxyacid oxidase (PDB entry 3sgz; Chen *et al.*, 2012 ▸) was used as the search model to solve the initial phase. Polypeptide models were built and refined with *REFMAC* (Murshudov *et al.*, 2011 ▸), *Coot* (Emsley *et al.*, 2010 ▸) and *PHENIX* (Afonine *et al.*, 2012 ▸). Detailed refinement statistics are presented in Table 1 ▸. Protein structures and electron-density maps were presented using *PyMOL* (http://www.pymol.org).

### 2.4. Site-directed mutagenesis   

The *hmo* (*orf22*) gene was amplified from *Amycolatopsis orientalis* genomic DNA by PCR amplification and was then subcloned into the expression vector pET-28a(+) to produce an N-terminally His_6_-tagged protein. The expression plasmid pET-28a and PCR products were digested with the restriction enzymes NdeI and XhoI, respectively, at 37°C for 5 h. DNA ligation was subsequently performed according to the manufacturer’s instructions (Novagen). The inserted gene of the clones was checked by restriction-enzyme analysis and justified by agarose electrophoresis and DNA sequencing. The site-directed point mutants Y128A, Y128C, Y128F, R163L, H252A and R255A were generated using QuikChange (Stratagene), in which the wild-type *hmo* gene was used as the template for single mutants. All mutants were confirmed by DNA sequencing. Mutant proteins were purified using the same protocol as used for recombinant wild-type (WT) Hmo.

### 2.5. Enzymatic activity assay   

Commercial α-hydroxyacids/α-ketoacids, including (*S*)-*p*-hydroxymandelate, (*S*)-mandelate, *p*-chloromandelic acid, *p*-bromomandelic acid, 2-chloromandelic acid, 4-(trifluoro­methyl)mandelate and benzylformate, were purchased from Sigma–Aldrich. Other unusual substrates, including α-hydroxy­amides/α-ketoamides, were chemically prepared following modified synthetic protocols (see below). A typical enzymatic reaction was carried out in HEPES buffer (20 m*M* HEPES, 100 m*M* NaCl pH 7.5) using the substrates mentioned above and protein (native Hmo or mutants) at 25°C for 4 h. After quenching with 6 *N* HCl, the filtered reaction solutions were subjected to a reverse-phase C_18_ column (4.6 × 250 mm, 5 µm, C_18_ Prodigy, Phenomenex) mounted on an Agilent 1260 Infinity Quaternary LC module in connection with ESI/MS (Thermo-Finnigan LTQ-XL), a Waters Alliance 2695 HPLC module with a Xevo TQ-S micro triple quadrupole mass spectrometer, or GC-MS (Thermo-Finnigan Polaris Q). The LC analytical mobile phase was programmed as water with 1% formic acid as solvent *A* and acetonitrile with 1% formic acid as solvent *B*, with a linear gradient from 2% to 40% solvent *B* in solvent *A* at a flow rate of 1.0 ml min^−1^ over 25 min followed by 98% solvent *B* for another 8 min. The analytes were monitored at a UV wavelength at 254 nm using both positive and negative modes for mass detection. LC-MS data were recorded and analyzed using the *MassLynx* or *Xcalibur* software.

### 2.6. Measurement of the production of hydrogen peroxide   

Hydrogen peroxide production by Hmo and its mutants was measured using the Fluorescent Hydrogen Peroxide/Peroxidase Detection Kit (Fluoro H_2_O_2_). We followed the general protocol provided by Cell Technology and detected the fluorescence to quantify the production of hydrogen peroxide (excitation at 579 nm and emission at 600 nm).

### 2.7. Hmo and Y128F mutant reactions in the presence of ^18^O_2_ and H_2_^18^O   

For the ^18^O_2_ experiments, the assay solution was made in a final volume of 200 µl that contained 20 m*M* HEPES, 100 m*M* NaCl, 2 m*M* (*S*)-mandelic acid and 0.4 mg Hmo Y128F mutant. The sample was subjected to vacuum and an N_2_ purge to remove O_2_. The sample was then refilled with ^18^O_2_. After 2 h of stirring at 25°C, the reaction mixture was denatured with 6 *N* HCl, centrifuged and analyzed by HPLC and GC-MS. For the H_2_^18^O experiment, the Hmo Y128F mutant assays were carried out with the same reactants in a final buffer solution of 90% H_2_^18^O. The enzymatic reactions were incubated at room temperature for 2 h and denatured with 6 *N* HCl. Both HPLC and GC-MS were used to detect the targets.

## 3. Results and discussion   

### 3.1. Reactivity of Hmo   

**Figure 2:**
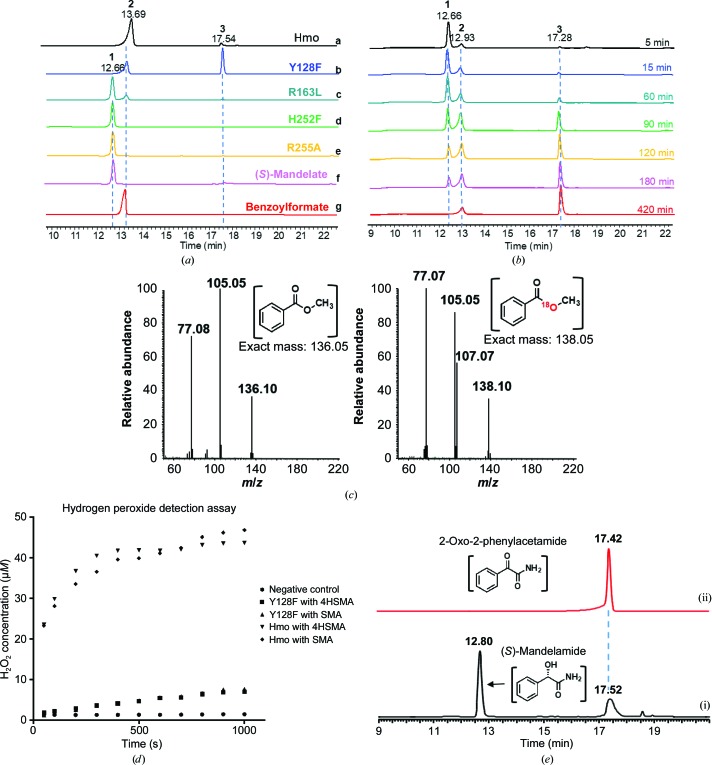
LC, UV and MS spectra for reactions catalyzed by Hmo and its mutants. (*a*) LC traces of enzymatic reactions with (*S*)-mandelate (**1**) as the substrate catalyzed by Hmo and its mutants: **a**, WT Hmo; **b**, Y128F mutant; **c**, R163L mutant; **d**, H252F mutant; **e**, R255A mutant; **f**, (*S*)-mandelate standard (**1**); **g**, benzoylformate standard (**2**). (*b*) Enzymatic reactions catalyzed by the Y128F mutant, where benzoate (**3**) is the major kinetic product over 420 min. (*c*) Mass spectra of methyl benzoate analyzed by GC-MS for enzymatic reactions conducted in the presence of H_2_^18^O (left) and ^18^O_2_ (right). (*d*) Formation of hydrogen peroxide (H_2_O_2_) in enzymatic reactions catalyzed by WT Hmo and the Y128F mutant in the presence of (*S*)-mandelate (SMA) or 4-­hydroxy-(*S*)-mandelate (4HSMA). (*e*) LC traces of enzymatic reactions catalyzed by the R163L mutant with α-(*S*)-mandelic amide as the substrate (i), in which the product is 2-oxo-2-phenylacetamide showing the same retention as the synthetic reference (ii).

We first set out to examine Hmo from *A. orientalis*. Biochemical analysis showed that Hmo efficiently transforms (*S*)-mandelate to benzoylformate (Hubbard *et al.*, 2000 ▸; Li *et al.*, 2001 ▸), while a small peak that emerged at 17.5 min on LC traces in a dose-dependent manner drew our attention [Fig. 2 ▸(*a*)]. This peak was determined to be benzoate by NMR and MS (Supplementary Fig. S3). The enantiopreference for substrates is for (*S*)-conformers, as (*R*)-mandelate was left unchanged (Supplementary Fig. S4). The reactivity of Hmo towards the substrates (*S*)-mandelate, l-*p*-HPG and (*S*)-2-methylphenylacetate showed that the first two are oxidized to benzoylformate with benzoate as a minor product, but the third cannot be oxidized. The utilization of l-*p*-HPG shows that Hmo can act as an amine oxidase. The *para* and *ortho* substituents on the phenyl ring of (*S*)-mandelate have little influence on the activity of the enzyme, as 4-chloro-, 2-chloro-, 4-bromo- and 4-trifluoromethyl-(*S*)-mandelate can all be oxidized by Hmo to the corresponding α-ketoacids with benzoate as a minor product (Supplementary Fig. S4; Supplementary Table S2). The α-hydroxyacid moiety is not limited to two carbons: (*S*)-3-phenyllactate can be oxidized to phenylpyruvate with phenylacetate as a minor product (Supplementary Fig. S4). Although minor decarboxylated acid products can be formed, our analysis agreed that Hmo is an oxidase [*k*_cat_ = 5.17 s^−1^, *K*_m_ = 0.16 m*M* for the conversion of (*S*)-mandelate to benzoylformate].

With respect to the mechanism of FMN-dependent oxidases, two mechanisms, a direct hydride-transfer mechanism (HT) and a carbanion mechanism (CA), have generally been referred to (Dijkman *et al.*, 2013 ▸; Walsh & Wencewicz, 2013 ▸). For HT, deprotonation of the α-OH by an active-site base yields an oxyanion species, which upon collapse transfers an α-hydride to FMN_ox_. For CA, the abstraction of a relatively acidic α-proton by an active-site base results in a dianionic/enolate intermediate, and electron transfer to this intermediate or covalent association with FMN_ox_ takes place. While the majority of relevant biochemical and kinetic studies support HT, unequivocal structural evidence remains lacking (Cao *et al.*, 2014 ▸; Dijkman *et al.*, 2013 ▸; Gillet *et al.*, 2016 ▸; Lederer *et al.*, 2016 ▸; Walsh & Wencewicz, 2013 ▸).

### 3.2. Model determination and overall structure   

**Figure 3:**
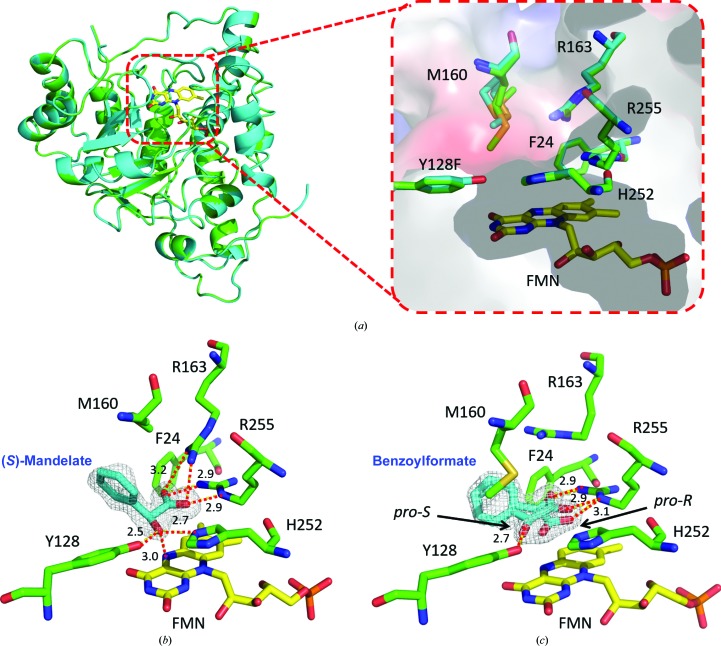
Liganded structures of Hmo. (*a*) Superposition of ternary complexes of wild-type Hmo on those of the Y128F mutant shows a low average root-mean-square deviation (r.m.s.d.) of 0.064 Å, where Hmo and the Y128F mutant are colored cyan and green, respectively. There is no significant difference between Hmo (green) and the Y128F mutant (cyan) in terms of the active-site geometry, except that the *p*-OH group is absent on the phenyl ring of Y128F. (*b*) When Hmo is bound by (*S*)-mandelate, Met160 flips away and Arg163 makes an electrostatic association with the carboxylic group of (*S*)-mandelate. (*c*) When benzoylformate is bound to Hmo, it adopts double conformations (*pro*-*S* or *pro*-*R*). Met160 stows back, while Arg163 flips away. The numbers are bond lengths in Å for the designated bonds. Free ligands, FMN and active-site residues are colored cyan, yellow and green, respectively. The 2*F*_o_ − *F*_c_ electron-density map is contoured at 2σ.

We then performed protein X-ray crystallography in an attempt to obtain snapshots of liganded structures in different states for Hmo and its Y128F mutant. The initial phase problem was solved by the molecular-replacement (MR) method using MDH and FCB2 as search models (Li *et al.*, 2007 ▸; Sukumar *et al.*, 2004 ▸). The solved structure of Hmo was used to generate other ligand-bound structures. The resolutions of the 18 structures ranged from 1.3 to 1.9 Å, all with good *R*_work_ and *R*_free_ values. (The diffraction parameters, refinement statistics and PDB codes are summarized in Table 1 ▸.) The structures of Hmo and its mutant with or without ligands all have a single polypeptide chain in the asymmetric unit, while a crystallo­graphic twofold axis generates a dimer. Gel-filtration chromatography and analytical ultracentrifugation analysis (Supplementary Fig. S6) also indicated that dimers are the most biologically relevant state for Hmo. Each monomer is made of a single (β/α)_8_-barrel domain, known as a TIM barrel, in which the C-terminal loops of the β1, β2 and β8 strands of the barrel constitute the substrate- and cofactor-binding sites [Fig. 3 ▸(*a*), Supplementary Fig. S7]. One tightly bound FMN cofactor is deeply buried inside the protein, while its redox-active isoalloxazine is accessible to the bulk solvent or substrate (Supplementary Fig. S7). Ternary complexes of the wild type or the Y128F mutant with (*S*)-*p*-hydroxy­mandelate or (*S*)-mandelate (substrates), oxidized mandelate [soaked with (*S*)-mandelate but oxidized to benzoylformate], *p*-hydroxy­benzoylformate or benzoylformate (products) or (*S*)-2-phenylpropionate (an analog/inhibitor) were obtained, in which each ligand fits well to the corresponding electron density [Figs. 3 ▸(*a*)–3 ▸(*c*) and 4 ▸(*a*)–4 ▸(*f*); Supplementary Figs. S8 and S9]. The physiological substrates other than enantiomers [for example (*R*)-mandelate], the competitive inhibitors and the products identified here were not anticipated, while these comprehensive complexes are likely to only be obtained in the case of Hmo and its mutants in the α-hydroxyacid oxidase family. This difficulty in and rarity of obtaining such complexes is commonly ascribed to the high substrate-turnover rates in flavin-dependent oxido­reductases (*k*_cat_/*K*_m_ > 10^5^ *M* ^−1^ s^−1^; too fast to retain their nascent states) even under flash-soaking/cryogenic cooling, as opposed to those of Hmo and its mutants, which are relatively slower by one order of magnitude (*k*_cat_/*K*_m_ ≃ 10^4^ *M* ^−1^ s^−1^).

### 3.3. Structural comparison and ligand-binding site   

Superposition of the ternary complexes with (*S*)-*p*-hydroxymandelate, (*S*)-mandelate, benzoylformate or (*S*)-2-phenylpropionate on the binary complex shows low average root-mean-square deviations (r.m.s.d.s) for 367 C^α^ backbone atoms, suggesting that there are no significant conformational changes when bound to or lacking a ligand [Fig. 3 ▸(*a*); Supplementary Fig. S10]. Some local structures do display conformational changes: Met160 near to the ligand entrance may serve as a gatekeeper at the entrance to the ligand-binding tunnel, where the closing/opening of the tunnel synchronizes with the absence/presence of a ligand [Fig. 3 ▸(*b*)]. Arg163 and Arg255 above and to the right of the isoalloxazine ring interact with the carboxylic end of an α-hydroxyacid through electrostatic forces. An α-ketoacid, however, makes no contact with Arg163, as the guanidino group flips 180° away [Figs. 3 ▸(*b*) and 3 ▸(*c*)]. The ligand-binding site is made up of six major residues (Phe24, Ala79, Tyr128, Met160, Arg163, His252 and Arg255) above the *si*-face (relative to the *sp* ^2^ N5) of the isoalloxazine ring [Figs. 3 ▸(*a*), 3 ▸(*b*) and 3 ▸(*c*)], where (*S*)-*p*-hydroxymandelate (substrate), (*S*)-mandelate (substrate) or (*S*)-2-phenylpropionate (inhibitor) all adopt a ‘V’ conformation with the carboxyl group orthogonal to the plane of the phenyl ring (Supplementary Figs. S8 and S9). Despite being enclosed by hydrophobic residues (Phe24, Ala79 and Tyr128), the cusp of the phenyl ring protrudes towards the funnel-like substrate entrance and is accessible to bulk solvent, allowing some extent of substrate promiscuity, which is consistent with the biochemical analysis [Fig. 3 ▸(*a*)]. The α-OH group is at low-barrier hydrogen-bond distances to Tyr128 (2.5 Å) and His252 (2.7 Å). Collectively, the α-hydroxyacid is anchored in place by four residues, Tyr128, Arg163, His252 and Arg255, where the α-H points towards N5 of the isoalloxazine ring at a distance of 3.0 Å, in line with the chirality of the reaction [Fig. 3 ▸(*b*)]. The importance of these residues was examined by site-directed mutagenesis, in which all mutants (R163A, H252F and R255A) except Y128F lost their activity. Moreover, the α-ketoacid and phenyl moieties of the product benzoylformate extend in a planar manner and sit above and in front of the isoalloxazine ring [Fig. 3 ▸(*c*)].

### 3.4. Catalytic mechanism   

**Figure 4:**
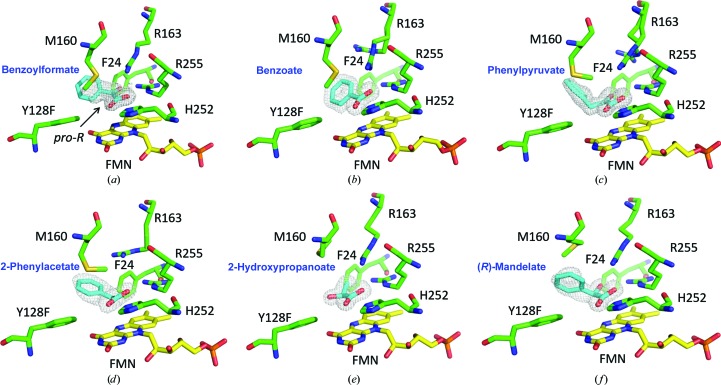
Ternary-complex structures of the Y128F mutant. (*a*) The Y128F mutant soaked with benzoylformate, which mainly adopts a *pro*-*R* conformation. (*b*) The Y128F mutant liganded with benzoate, which is the product of the four-electron oxidation reaction catalyzed by the Y128F mutant in the presence of (*S*)-mandelate. (*c*) The Y128F mutant liganded with phenylpyruvate, an oxidized product, when soaked with phenyllactate. (*d*) The Y128F mutant liganded with phenylacetate, which is the product of the four-electron oxidation reaction catalyzed by the Y128F mutant in the presence of 2-­phenyllactate. (*e*) Y128F bound by the α-hydroxylacid analog 2-hydroxypropanoate. (*f*) When the Y128F mutant is bound by (*R*)-mandelate, the structural conformation is similar to that of (*S*)-mandelate, while the α-OH group points away from His252. The free ligands, FMN and active-site residues are shown in cyan, yellow and green, respectively. The 2*F*_o_ − *F*_c_ electron-density map is contoured at 2σ.

**Figure 5:**
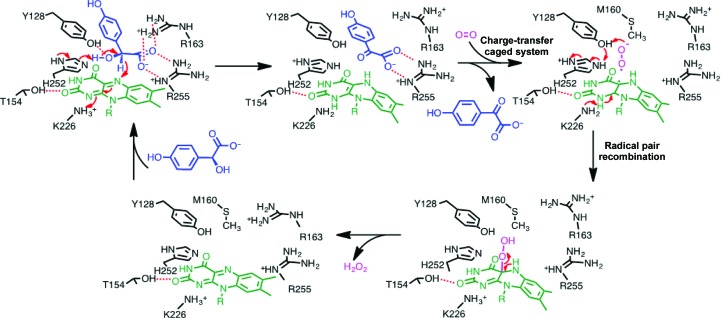
The catalytic cycle of Hmo in the conversion of (*S*)-mandelate to benzoylformate with the concomitant formation of H_2_O_2_.

Tyr128 and His252 in the active site are highly conserved in the family of FMN-dependent α-hydroxyacid oxidases, in which they often serve as a catalytic dyad (Supplementary Fig. S2). The spatial arrangement of the substrate (*S*)-mandelate and the cofactor FMN in the crystal structures of Hmo favors the HT mechanism, because the distance between the N^∊^ atom of His252 and α-OH (2.5 Å) is shorter than that between the N^∊^ atom and C′^α^ (3.0 Å). Crystals soaked with the inhibitors (*S*)-2-phenylpropionate or (*R*)-mandelate also show a similar distance (3.1 Å) between N^∊^ and C′^α^ as that for (*S*)-mandelate but are devoid of reactivity [Fig. 4 ▸(*f*), Supplementary Fig. S9]. In this context, His252 is likely to act as the base deprotonating α-OH to form an oxyanion that is stabilized by Tyr128 (Dubois *et al.*, 1990 ▸; Gondry *et al.*, 2001 ▸). Upon collapse of the oxyanion, an α-hydride is expelled to FMN in a hydrophobic chamber at a short distance, fulfilling the reductive half-reaction. FMN_ox_ is regenerated by relaying one electron from FMN_red_ to O_2_, forming a superoxide–FMN semiquinone radical pair, prior to its release as H_2_O_2_ in the oxidative half-reaction to complete one round of the catalytic cycle (Fig. 5 ▸; Massey, 1995 ▸; Mattevi, 2006 ▸). While the crystals were soaked with (*S*)-mandelate, the substrate was converted to benzoylformate. The product unexpectedly adopts two different spatial orientations, *pro*-*S* or *pro*-*R*, pointing towards or away from the *si* face of the isoalloxazine ring, respectively [Fig. 3 ▸(*c*)]. Both the *pro*-*S* and *pro*-*R* orientations have a similar coplanar conformation without interacting with Arg163. However, when crystals were soaked with benzoylformate the *pro*-*R* orientation was almost always observed (Supplementary Fig. S11). We reasoned that the *pro*-*S* product is initially formed and that it then converts to the thermodynamically more favorable *pro*-*R* orientation. This may be owing to a short distance between the α-keto group of *pro*-*S* benzoylformate and *p*-OH of Tyr128 and N^∊^ of His252 (2.3 and 2.8 Å, respectively) as a result of the van der Waals repulsion force, thus facilitating the release of benzoylformate (the first two-electron oxidation product) from the binding site [Fig. 3 ▸(*c*)]. In the Y128F mutant, the efficiency of releasing the product from the active site may be somewhat hampered.

### 3.5. Conversion from oxidase to oxidative decarboxylase   

Two mutants, Y128F and H252F, were subjected to a biochemical examination. The latter (H252F) showed no detectable activity, consistent with its designated role, whereas the former (Y128F) showed the activity of an LMO-like enzyme and catalytically converted (*S*)-mandelate to benzoate [Figs. 1 ▸(*d*) and 2 ▸(*b*)]. In detail, no products (benzoate and H_2_O_2_) were detected when the wild type (WT) or Y128F mutant was assayed in an anaerobic (no O_2_) condition containing (*S*)-mandelate or benzoylformate, suggesting that O_2_ is the prerequisite electron acceptor in the oxidation reaction. Despite the fact that H_2_O_2_ can act as either an oxidant or a reductant (Lopalco *et al.*, 2016 ▸), there was still no product under the same reaction conditions with the addition of H_2_O_2_ (2 h, pH 7.3, 2 m*M* H_2_O_2_ with the WT or the Y128F mutant). No product was again detected when benzoylformate was incubated aerobically (with O_2_) with the WT or the Y128F mutant plus H_2_O_2_. As a result, benzoate can only be formed from (*S*)-mandelate under aerobic conditions, despite benzoylformate and H_2_O_2_ being the apparent intermediate and oxidant, respectively. We thus speculated that the intermediate, the oxidant and FMN ought to associate in a well organized manner to allow the second two-electron oxidation to proceed. To prove this, we performed a stable isotope-labeling experiment. Using (*S*)-mandelate, H_2_^18^O_2_ and the Y128F mutant under an anaerobic condition, no benzoate was detected. In contrast, ^18^O-benzoate was detected when the reactions were performed under the same condition with ^18^O_2_ in lieu of H_2_^18^O_2_ [Fig. 2 ▸(*c*)]; thus, we conclude that free H_2_O_2_ is not the effective oxidant.

The conversion rates of (*S*)-mandelate to benzoylformate or to benzoate by the Y128F mutant were estimated: *k*_cat_ = 0.05 s^−1^, *K*_m_ = 0.13 m*M* and *k*_cat_ = 4.5 s^−1^, *K*_m_ = 0.15 m*M*, respectively. The *K*_m_ values are comparable, suggesting that the substrate-binding affinity of the WT or the Y128F mutant is similar, but the latter specializes in four-electron oxidation. The binding affinity of benzoylformate for Hmo or the Y128F mutant was further determined by ITC. In view of the binding affinity, the discrepancy between the affinity of benzoyl­formate for Hmo (*K*_d_ = 1.9 m*M*) and that of benzoylformate for the Y128F mutant (*K*_d_ = 1.4 m*M*) is marginal, with the latter being slightly stronger than the former (Supplementary Fig. S5), suggesting that the single mutation has little influence but allows benzoylformate to better interact with the Y128F mutant. The level of H_2_O_2_ in reactions with the Y128F mutant is inverse to the level of benzoate, in contrast to those with the WT, in which the level of H_2_O_2_ is proportional to that of benzoylformate [Fig. 2 ▸(*d*)]. R163L, a low-activity mutant, was further assayed against α-(*S*)-mandelamide [2-(*S*)-hydroxy-2-phenylethylamide]. This substrate can be recognized and oxidized to the α-ketoamide by the R163L mutant, but not to benzoate [Fig. 2 ▸(*e*)], indicating that the timing of the decarboxylation step is critical to the four-electron oxidation. In brief, one O atom from O_2_ is incorporated into benzoate, where the peroxide is not in the form of free H_2_O_2_ in solution, but in a well organized manner in which the α-ketoacid and FMN_red_ engage in the oxidative decarboxylation reaction, well beyond the non-ping-pong mechanism described for LMO (Choong & Massey, 1980 ▸; Ghisla & Massey, 1977 ▸; Lockridge *et al.*, 1972 ▸; Walsh *et al.*, 1973 ▸). As revealed above, the *pro*-*R* orientation instead of the *pro*-*S* is in line with the trajectory of the attack of C4α-peroxide (a Baeyer–Villiger-type reaction) or the nucleophilic *sp* ^3^ N5 of FMN_red_ on the α-keto carbonyl C atom of the α-ketoacid, implying that stabilization of the peroxide intermediate and swift reorientation of the products are possible reasons for the conversion of the Y128F mutant from an oxidase to an oxidative decarboxylase [Fig. 4 ▸(*a*)] (Lyu *et al.*, 2019 ▸).

## 4. Conclusions   

While flavoproteins, which are omnipresent in all living organisms, have been studied for over a century, many outstanding issues remain unresolved. In this study, structural biology in conjunction with biochemical examinations allowed us to dissect the oxidation reactions carried out by Hmo in steps. In the two-electron oxidation reaction, FMN_ox_ is reduced by a hydride directly from an α-hydroxyacid; FMN_red_ is instantly oxidized by O_2_ to yield H_2_O_2_. The collection of multiple liganded snapshots of phases in the active site tangibly supports the HT mechanism and meanwhile accounts for some extent of substrate promiscuity. In the Y128F mutant, the two-electron oxidation reaction is catalytically extended to a four-electron oxidation reaction. The rationale for the reaction is proposed on the basis of the structural information obtained as follows: the absence of the *p*-OH group in the Y128F mutant substantiates the peroxide anion adduct as a nucleophilic group, and the reorientation of the α-ketoacid in the *pro*-*R* configuration above the isoalloxazine ring in the Y128F mutant collaterally provides a better attacking trajectory for nucleophilic FMN_red_ or C4α-peroxide. The Y128F mutant is thereby transformed into a monooxygenase-like enzyme that catalyzes the oxidative decarboxylation reaction, in which the phenolic O atom of Tyr128 in Hmo apparently plays a pivotal role in control of the oxidative cascade through activating and stabilizing reactants and intermediates in addition to facilitating the release of products.

## Supplementary Material

Supplementary materials and methods, figures and tables. DOI: 10.1107/S2059798319009574/ag5030sup1.pdf

PDB reference: *p*-hydroxymandelate oxidase, 5zzp

PDB reference: complex with 4-hydroxy-(*S*)-mandelate, 5zzq

PDB reference: complex with (*S*)-mandelate, 5zzr

PDB reference: complex with benzoate, 5zzs

PDB reference: complex with 2-phenylpropanoate, 6a00

PDB reference: complex with benzoylformate, 6a08

PDB reference: Y128F mutant, 6a13

PDB reference: complex with (*R*)-mandelate, 5zzx

PDB reference: complex with 2-hydroxypropanoate, 5zzy

PDB reference: complex with 2-phenylpropanoate, 6a0d

PDB reference: complex with 2-hydroxy-3-phenylpropanoate, 6a0g

PDB reference: complex with 2-phenylacetate, 6a0m

PDB reference: complex with benzaldehyde, 6a0o

PDB reference: complex with (*S*)-mandelate, 6a0v

PDB reference: complex with benzoate, 6a0y

PDB reference: complex with phenylpyruvic acid, 6a11

PDB reference: complex with benzoylformate, 6a19

PDB reference: complex with 4-hydroxy-(*S*)-mandelate, 6a1a

## Figures and Tables

**Table:** Values in parentheses are for the highest resolution shell. 4HSMA, 4-hydroxy-(*S*)-mandelate; SMA, (*S*)-mandelate; BF, benzoylformate; BA, benzoate; SPPA, 2-­phenylpropanoate; BZ, benzaldehyde; PA, 2-phenylacetate; PLA, 2-hydroxy-3-phenylpropanoate; LLAC, 2-hydroxypropanoate; RMA, (*R*)-mandelate; PPY, phenylpyruvic acid.

	Hmo	Hmo–4HSMA	Hmo–SMA	Hmo–BF	Hmo–BA	Hmo–SPPA	Y128F	Y128F–BZ	Y128F–LLAC
PDB code	5zzp	5zzq	5zzr	6a08	5zzs	6a00	6a13	6a0o	5zzy
Data collection									
Wavelength (Å)	1.0	1.0	1.0	1.0	1.0	1.0	1.0	1.0	1.0
Space group	*I*422	*I*422	*I*422	*I*422	*I*422	*I*422	*I*422	*I*422	*I*422
*a*, *b*, *c* (Å)	137.9, 137.9, 112.3	137.8, 137.8, 111.3	137.7, 137.7, 111.6	137.9, 137.9, 111.8	137.9, 137.9, 111.8	138.2, 138.2, 111.7	137.5, 137.5, 112.0	137.7, 137.7, 112.0	137.8, 137.8, 111.9
α, β, γ (°)	90, 90, 90	90, 90, 90	90, 90, 90	90, 90, 90	90, 90, 90	90, 90, 90	90, 90, 90	90, 90, 90	90, 90, 90
Resolution range (Å)	30–1.39 (1.44–1.39)	30–1.32 (1.37–1.32)	30–1.31 (1.36–1.31)	30–1.55 (1.61–1.55)	30–1.40 (1.45–1.40)	30–1.60 (1.66–1.60)	30–1.70 (1.76–1.70)	30–1.65 (1.71–1.65)	30–1.50 (1.55–1.50)
*R*_merge_ † (%)	3.7 (73.0)	3.8 (80.1)	3.7 (67.0)	4.0 (79.0)	3.9 (67.1)	4.8 (66.2)	3.5 (69.2)	3.9 (62.1)	5.4 (82.1)
〈*I*/σ(*I*)〉	36.1 (2.4)	43.5 (2.5)	34.5 (2.3)	43.1 (2.9)	32.3 (2.9)	28.5 (2.1)	41.3 (3.1)	31.0 (2.2)	31.2 (2.0)
Completeness (%)	99.9 (100.0)	100.0 (100.0)	99.0 (100.0)	100.0 (100.0)	99.0 (99.7)	99.5 (97.9)	100.0 (100.0)	100.0 (100.0)	99.9 (99.1)
Multiplicity	9.4 (9.4)	12.2 (12.0)	9.8 (9.5)	12.8 (11.2)	9.7 (9.5)	8.8 (8.0)	11.2 (11.1)	9.5 (8.7)	11.6 (8.3)
Refinement									
Resolution range (Å)	30–1.39 (1.44–1.39)	30–1.32 (1.37–1.32)	30–1.31 (1.36–1.31)	30–1.55 (1.61–1.55)	30–1.40 (1.45–1.40)	30–1.60 (1.66–1.60)	30–1.70 (1.76–1.70)	30–1.65 (1.71–1.65)	30–1.50 (1.55–1.50)
*R*_work_ ‡ (%)	17.0 (28.0)	17.5 (23.7)	16.6 (22.9)	16.9 (22.0)	16.4 (23.1)	17.4 (26.8)	17.8 (24.2)	15.8 (20.6)	15.8 (25.6)
*R*_free_ § (%)	18.3 (30.0)	18.8 (25.3)	17.7 (22.3)	17.9 (20.1)	18.4 (25.0)	19.0 (30.8)	20.0 (26.2)	18.2 (22.1)	16.8 (25.8)
R.m.s. deviations									
Bond lengths (Å)	0.010	0.010	0.009	0.008	0.015	0.010	0.018	0.010	0.023
Bond angles (°)	1.41	1.42	1.41	1.26	1.37	1.223	1.490	1.446	2.119
No. of reflections	100548	112024	112497	75720	102688	69702	58599	54457	78351
No. of atoms									
Protein	2785	2817	2622	2684	2656	2651	2510	2659	2551
Ligand/ion	31	43	53	65	58	53	31	58	37
Water	403	321	402	329	368	307	316	364	341
*B* factors (Å^2^)									
Protein	18.2	19.5	19.7	19.1	18.2	19.8	18.6	17.7	18.4
Ligand/ion	10.7	15.6	19.0	20.6	18.9	26.9	10.8	19.4	14.6
Water	32.4	30.1	33.9	31.7	32.8	31.6	32.1	32.6	32.2

**Table:** 

	Y128F–RMA	Y128F–4HSMA	Y128F–SMA	Y128F–BF	Y128F–BA	Y128F–PPY	Y128F–PA	Y128F–PLA	Y128F–SPPA
PDB code	5zzx	6a1a	6a0v	6a19	6a0y	6a11	6a0m	6a0g	6a0d
Data collection									
Wavelength (Å)	1.0	1.0	1.0	1.0	1.0	1.0	1.0	1.0	1.0
Space group	*I*422	*I*422	*I*422	*I*422	*I*422	*I*422	*I*422	*I*422	*I*422
*a*, *b*, *c* (Å)	138.1, 138.1, 112.4	137.8, 137.8, 111.5	137.8, 137.8, 111.8	137.9, 137.9, 112.3	137.7, 137.7, 112.0	138.5, 138.5, 111.5	138.2, 138.2, 111.2	138.3, 138.3, 111.4	137.9, 137.9, 111.3
α, β, γ (°)	90, 90, 90	90, 90, 90	90, 90, 90	90, 90, 90	90, 90, 90	90, 90, 90	90, 90, 90	90, 90, 90	90, 90, 90
Resolution range (Å)	30–1.50 (1.55–1.50)	30–1.35 (1.40–1.35)	30–1.39 (1.44–1.39)	30–1.55 (1.61–1.55)	30–1.65 (1.71–1.65)	30–1.45 (1.50–1.45)	30–1.75 (1.81–1.75)	30–1.80 (1.86–1.80)	30–1.65 (1.71–1.65)
*R*_merge_ † (%)	6.3 (78.0)	5.1 (74.6)	4.3 (73.1)	3.6 (75.0)	3.9 (62.1)	4.1 (79.0)	3.7 (79.0)	4.8 (81.0)	3.5 (71.0)
〈*I*/σ(*I*)〉	27.5 (2.0)	36.09 (2.7)	41.0 (2.9)	32.9 (2.7)	31.0 (2.2)	42.0 (2.7)	35.8 (2.0)	34.5 (2.7)	35.9 (2.4)
Completeness (%)	99.8 (100.0)	100.0 (100.0)	100.0 (100.0)	99.9 (100.0)	100.0 (100.0)	99.9 (100.0)	99.9 (100.0)	99.9 (100.0)	99.9 (100.0)
Multiplicity	9.6 (9.3)	12.2 (12.0)	12.2 (12.0)	9.6 (9.2)	9.5 (8.7)	11.8 (11.9)	9.3 (7.8)	11.1 (10.7)	9.7 (9.2)
Refinement									
Resolution range (Å)	30–1.50 (1.55–1.50)	30–1.35 (1.40–1.35)	30–1.39 (1.44–1.39)	30–1.55 (1.61–1.55)	30–1.65 (1.71–1.65)	30–1.45 (1.50–1.45)	30–1.75 (1.81–1.75)	30–1.80 (1.86–1.80)	30–1.65 (1.71–1.65)
*R*_work_ ‡ (%)	17.5 (30.7)	16.7 (23.5)	16.7 (22.9)	15.7 (24.4)	15.8 (20.6)	16.9 (23.9)	16.4 (24.1)	16.0 (22.9)	11.7 (23.3)
*R*_free_ § (%)	19.9 (32.7)	18.0 (24.6)	18.4 (23.1)	17.3 (26.6)	18.2 (22.1)	19.1 (24.4)	18.5 (26.5)	18.2 (25.6)	19.1 (25.2)
R.m.s. deviations									
Bond lengths (Å)	0.023	0.008	0.008	0.009	0.010	0.016	0.018	0.021	0.022
Bond angles (°)	2.121	1.3811	1.3361	1.460	1.446	1.433	1.834	2.044	2.080
No. of reflections	80736	106077	98014	74156	54457	91944	49594	46666	59471
No. of atoms									
Protein	2547	2640	2636	2703	2659	2677	2555	2545	2530
Ligand/ion	42	55	53	53	58	43	41	55	53
Water	369	401	400	400	364	357	279	288	304
*B* factors (Å^2^)									
Protein	17.7	16.8	16.9	21.9	17.7	20.0	25.8	25.3	20.2
Ligand/ion	25.9	18.1	17.8	29.4	19.4	20.1	26.2	23.2	27.5
Water	33.1	33.6	32.2	37.3	32.6	34.9	37.0	36.7	33.6

† *R*_merge_ = 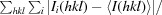 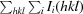, where *I_i_*(*hkl*) is the average intensity value of equivalent reflections.

‡ *R*_work_ = 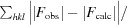 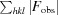.

§ *R*_free_ was calculated from 5% of data that were randomly excluded from refinement.
