# Supplementary material for: Biochemical and structural explorations of α-hydroxyacid oxidases reveal a four-electron oxidative decarboxylation reaction
Source: Acta Crystallogr D Struct Biol. 2019 Jul 30;75(Pt 8):733–42. doi: 10.1107/S2059798319009574 (PMC6677016; doi:10.1107/S2059798319009574)
Supplement: Supplementary file 1 [file d-75-00733-sup1.pdf]

STRUCTURAL BIOLOGY

Volume 75 (2019)

Supporting information for article:

Biochemical and structural explorations of α-hydroxyacid oxidases reveal a four-electron oxidative decarboxylation reaction

Hsien-Wei Yeh, Kuan-Hung Lin, Syue-Yi Lyu, Yi-Shan Li, Chun-Man Huang, Yung-Lin Wang, Hao-Wei Shih, Ning-Shian Hsu, Chang-Jer Wu and Tsung-Lin Li

S1. Materials and Methods

S1.1. Cloning and site-directed mutagenesis

The *hmo* (*orf22*) gene was amplified from *Amycolatopsis orientalis* genomic DNAs by PCR amplification and then sub-cloned into the expression vector pET-28a (+) to provide an N-terminal His_6-tagged protein. The expression plasmids pET-28a and PCR products were respectively digested with restriction enzymes NdeI and XhoI at 37°C for 5 hrs. DNA ligation was subsequently performed according to the manufacturers' instruction (Novagene). The inserted gene of clones was checked by restriction-enzyme analysis justified by agarose electrophoresis and DNA sequencing. Site-directed point mutation mutants Y128A, Y128C, Y128F, R163L, H252A and R255A were made using QuickChange (Stratagene), where the wild-type *hmo* was used as the template for single mutation mutants. All mutants were confirmed by DNA sequencing. Mutant proteins were purified with the same protocol as recombinant wild-type Hmo (see below). Primers used in this study are listed in **Table S1**.

S1.2. Compound characterization and synthesis

All chemically synthesized compounds were purified using column chromatography and characterized by MS or NMR for enzymatic experiments unless otherwise stated. ^{1}H and ^{13}C NMR spectra in DMSO-d_6 or CD_3OD for selected compounds were acquired from Bruker Avance 600 spectrometers equipped with CryoProbe™, and chemical shifts of ^{1}H NMR spectra were reported in units of ppm relative to tetramethylsilane (TMS). The TopSpin (version 3.5) program was used to process NMR data sets and detailed peak information (chemical shift δ; peak multiplicity; coupling constant *J*; proton number) was presented as follows: (refer to Figure S15-16 for NMR spectra):

S1.2.1. Synthesis of (*S*)-mandelamide

For the synthesis of (*S*)-mandelamide, the (*S*)-mandelic acid (1.5 g, 10 mmol) was dissolved in MeOH; acetyl chloride (1.5 mL, 2.47 g, 31.4 mmol) was added dropwise in ice bath at 0-4°C. After stirring the solution for 4 hrs, ammonium hydroxide (20 mmol) was added and mixed for another 2 hrs. The reaction mixture was dried and re-dissolved with ethyl acetate (10 mL) for further purification by flash column chromatography (hexane:EtOAc = 4:1 (v/v)) to afford 0.9 g of the titled compound (65% yield) as a white powder. LC-MS: m/z 150.4. $[M-H]^-$. ^{1}H-NMR of (*S*)-mandelamide (600 MHz, CD_3OD): δ 5.00 (*s*, 1H), 7.29 (*m*, 1H), 7.34 (*m*, 2H), 7.47 (*dd*, J = 7.2, 1.8, 1H); ^{13}C NMR of (*S*)-mandelamide (600 MHz, CD_3OD): δ 75.5 (C2), 128.1 (C4, C8), 129.2 (C5, C7), 129.5 (C6), 141.9 (C3), 178.8 (C1) (Fig. S13).

S1.2.2. Synthesis of 2-oxo-2-phenylacetamide

Benzoylformic acid (1, 3 g, 25 mmol) and acetyl chloride (1.5 mL, 2.47 g, 19.5 mmol) were added in anhydrous MeOH (5 mL). The reaction mixture was stirred and bathed in ice to keep 0-4°C for 6 hrs. The solvent was evaporated and then dissolved in MeOH again (20 mL); ammonium hydroxide (20 mmol) was subsequently added. The solution was stirred at low temperature for another 2 hrs. The products were purified by column chromatography (EtOAc:hexane = 1:4 (v/v), 70% overall yield). LC-MS: m/z 148.3. $[M-H]^-$. ^{1}H-NMR of 2-oxo-2-phenylacetamide (600 MHz, CD_3OD): δ 7.33 (*m*, 1H), 7.54 (*t*, J = 7.8, 2H), 7.66 (*m*, 1H), 8.09 (*dd*, J = 6.6, 1.8, 2H); ^{13}C NMR of 2-oxo-2-phenylacetamide (600 MHz, CD_3OD): δ 129.5 (C4, C8), 131.4 (C5, C7), 134.5 (C3), 135.7 (C6), 169.2 (C1), 191.3 (C2) (Fig. S14).

S1.3. Analytical ultracentrifuge analysis (AUC)

A Beckman-Coulter XL-I analytical ultracentrifuge was used for the sedimentation velocity analysis of the samples. For determining particle-size distribution of our proteins, 400 μL of a buffer solution (50 mM HEPES, pH 8) with 1 mg/mL sample were loaded in the sample cell of 12-mm standard double-sector Epon charcoal-filled centerpieces and placed in an An-60 Ti rotor. The measurement was run at a constant rotation speed of 40,000 rpm at 20°C. Absorbance (280 nm) signals were collected in parallel in the same run every 3 mins for 6 hrs. The raw data (ex. the density and viscosity of sample/buffer) of experiments were calculated using the SedFit software (http:// www.jphilo.mailway.com /default.htm). All samples were visually checked for clarity after ultracentrifugation, and no indication of precipitation was observed.

S1.4. Binding affinity measurement (isothermal titration calorimetry, ITC)

Calorimetric titrations of Hmo or mutant Y128F with benzoylformate were performed on a microcalorimeter (MicroCal iTC200) at 25°C. Proteins were dialysis with a 50 mM HEPES buffer solution at pH 8.0 before analysis. The sample cell (200 μL) and injection syringe (40 μL) were filled with 1 μM protein solution and 80 mM benzoylformate, respectively. A typical experiment includes 18 injections. The data were analyzed by using Origin (V. 7.0), where heat areas were integrated and fitted with a standard single-site binding model.

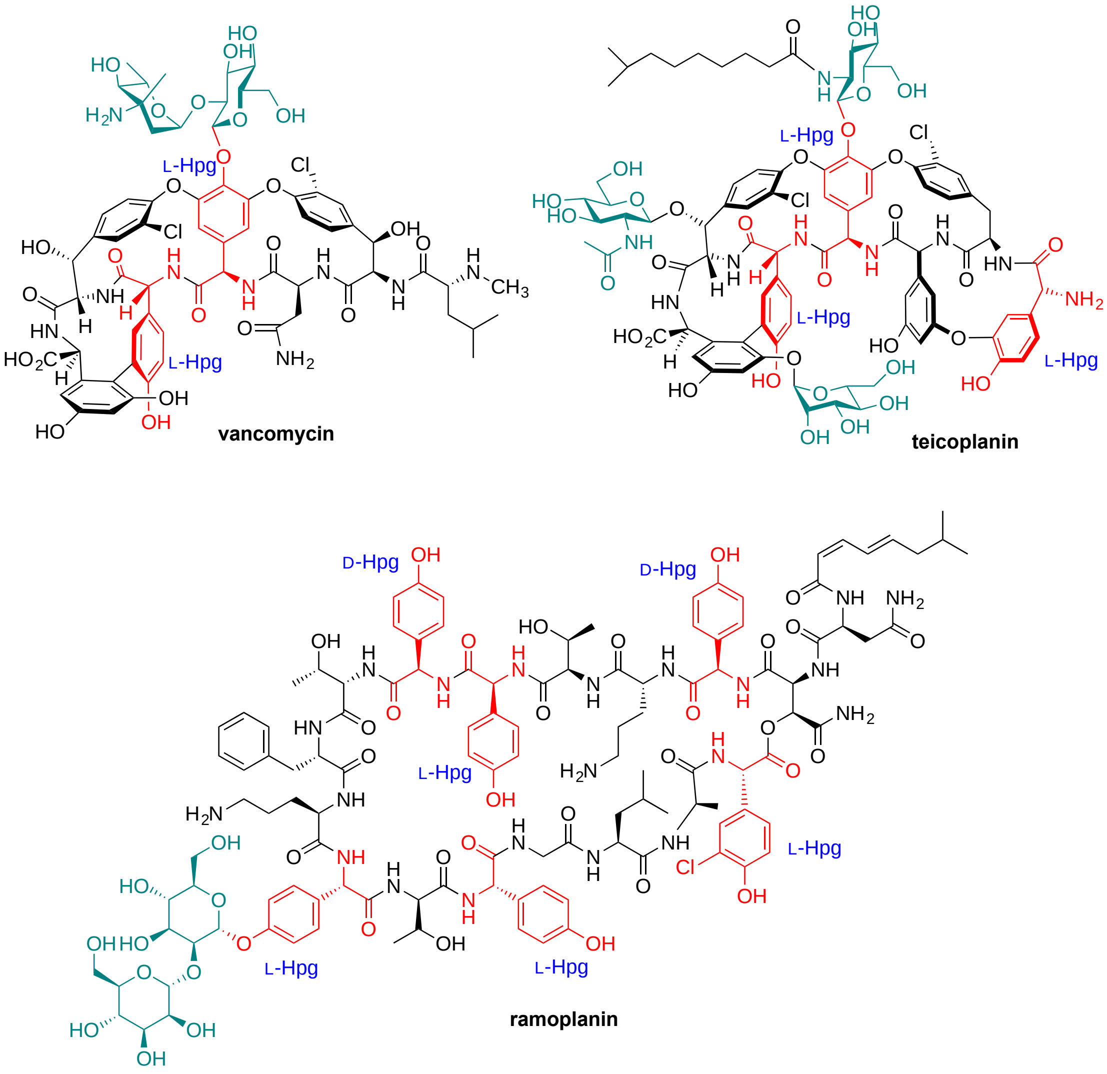

Figure S1 Clinically important glycopeptide natural products. The backbones of vancomycin, teicoplanin and ramoplanin are composed of various numbers of α-amino-acid building units, including non-proteinogenic *p*-OH-phenylglycine (*p*-HPG) colored red.

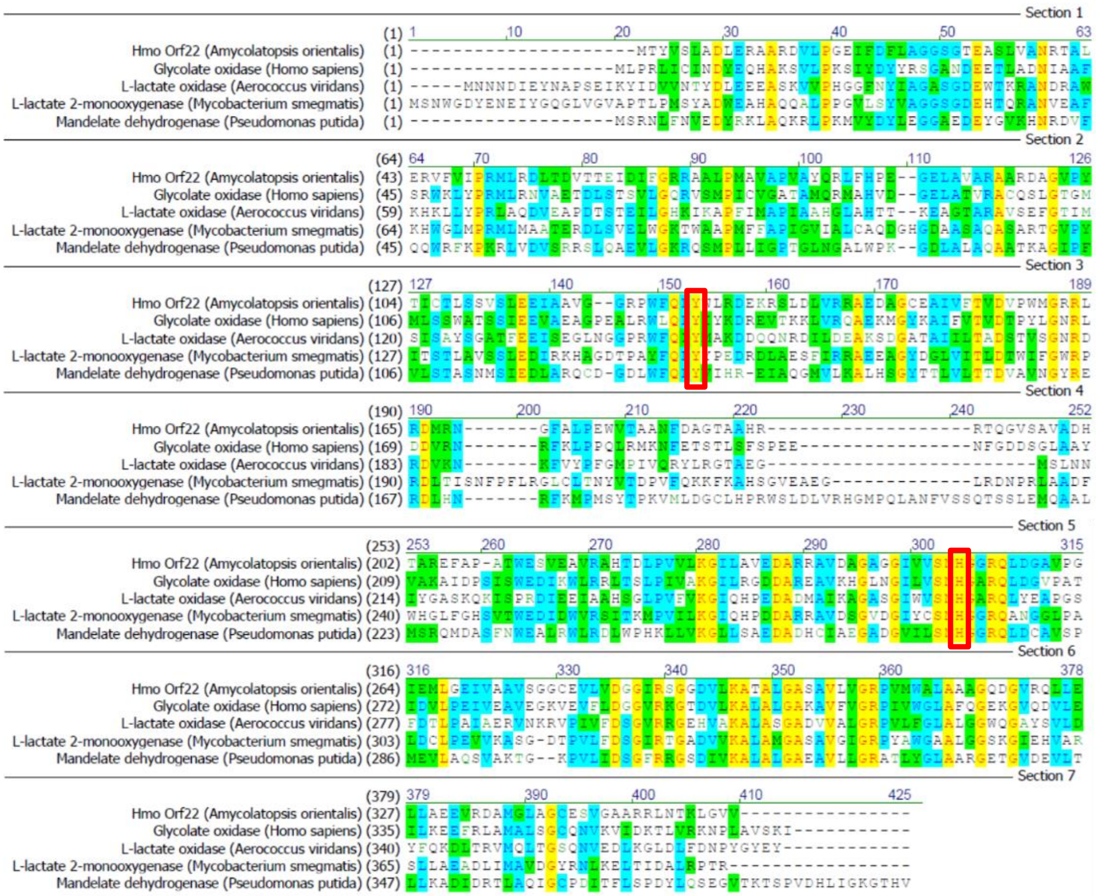

Figure S2 Protein sequence alignment of Hmo and homologous FMN oxidoreductases. The amino acid sequence alignment of FMN oxidoreductases, including Hmo (*Amycolatopsis orientalis* cosmid PCZA361, sequence ID: CAA11762.1) and representative homologous proteins from *Aerococcus viridans* (sequence ID: WP_003142047.1), *Mycobacterium smegmatis* (sequence ID: WP_003895417.1), *Pseudomonas putida* (sequence ID: WP_019438745.1) and *Homo sapiens* (sequence ID: NP_060015.1). Strictly conserved amino acids are highlighted in yellow, which mainly are residues around the FMN binding site. The typical catalytic dyad (Tyr and His) is framed in red.

A

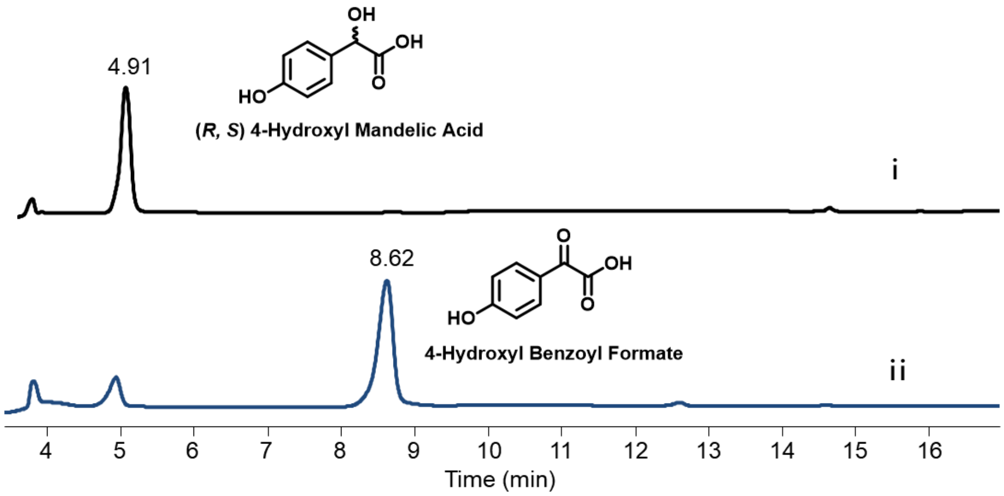
4.91
OH
OH
HO
O
(R, S) 4-Hydroxyl Mandelic Acid
i
8.62
O
OH
HO
O
4-Hydroxyl Benzoyl Formate
ii
4 5 6 7 8 9 10 11 12 13 14 15 16
Time (min)

B

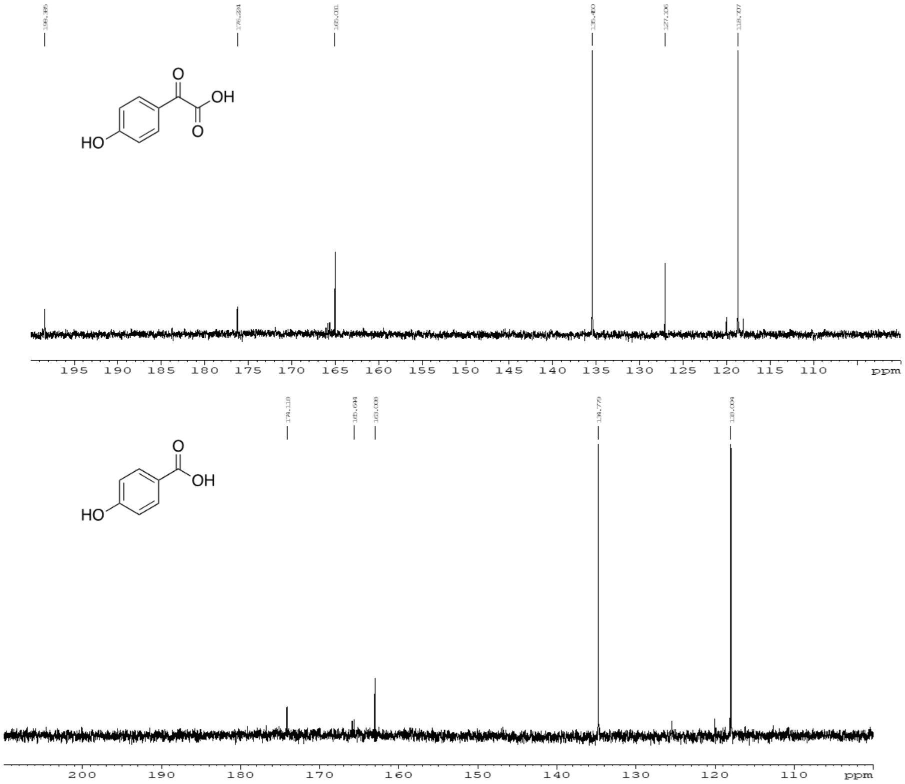
O
OH
HO
O
195 190 185 180 175 170 165 160 155 150 145 140 135 130 125 120 115 110 ppm
O
OH
HO
200 190 180 170 160 150 140 130 120 110 ppm

C

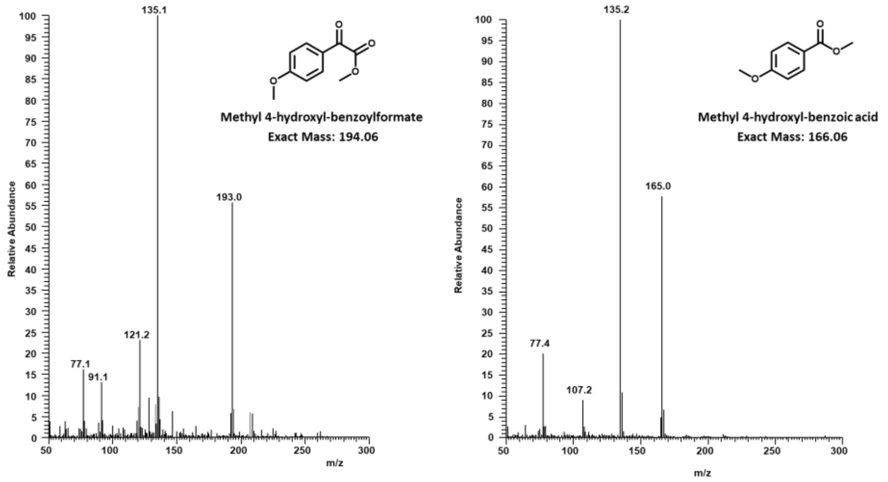

Figure S3 LC, MS and NMR spectra for the products *p*-OH-benzoylformate and *p*-OH-benzoate converted from the substrate (*S*)-*p*-OH mandelate in an enzymatic reaction catalyzed by Hmo. (A) The LC traces of enzymatic reactions catalyzed by Hmo, where the racemic substrate *p*-OH mandelate is shown in trace I; the unconsumed substrate (*R*)-*p*-OH mandelate, and reaction products *p*-OH-benzoylformate (major) and *p*-OH-benzoate (minor) are shown in trace ii. (B) ^{13}C NMR spectra of *p*-OH-benzoylformate (top panel) and *p*-OH-benzoate (bottom panel). (C) Electron impact (EI) mass spectra of methylated *p*-OH-benzoylformate (left panel) and *p*-OH-benzoate (right panel).

A

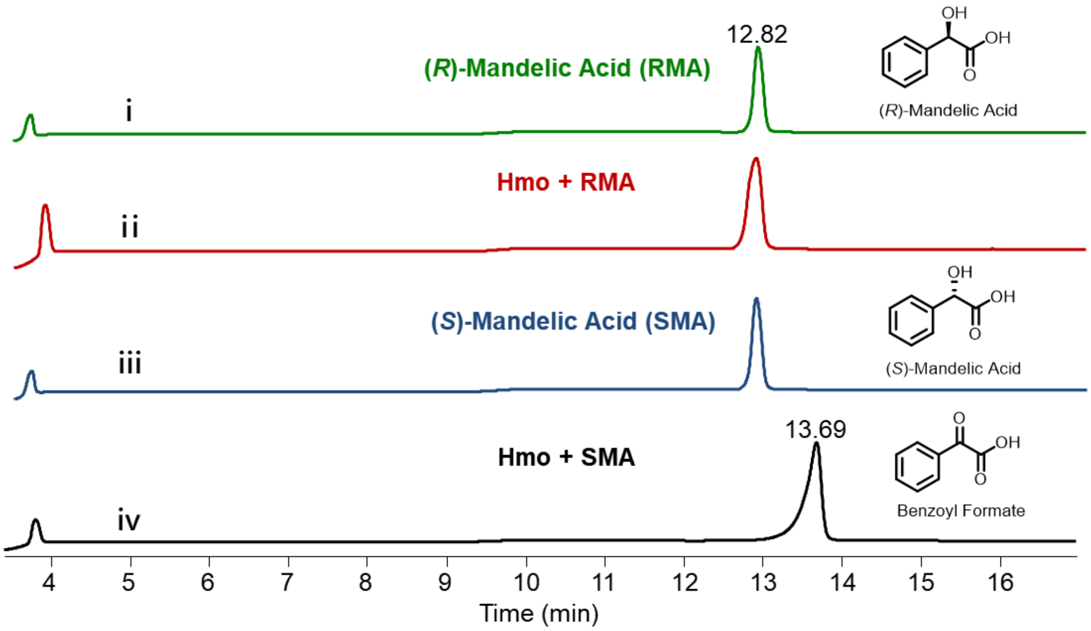
12.82
(R)-Mandelic Acid (RMA)
(R)-Mandelic Acid
i
Hmo + RMA
ii
(S)-Mandelic Acid (SMA)
(S)-Mandelic Acid
iii
13.69
Hmo + SMA
Benzoyl Formate
iv
4 5 6 7 8 9 10 11 12 13 14 15 16
Time (min)

B

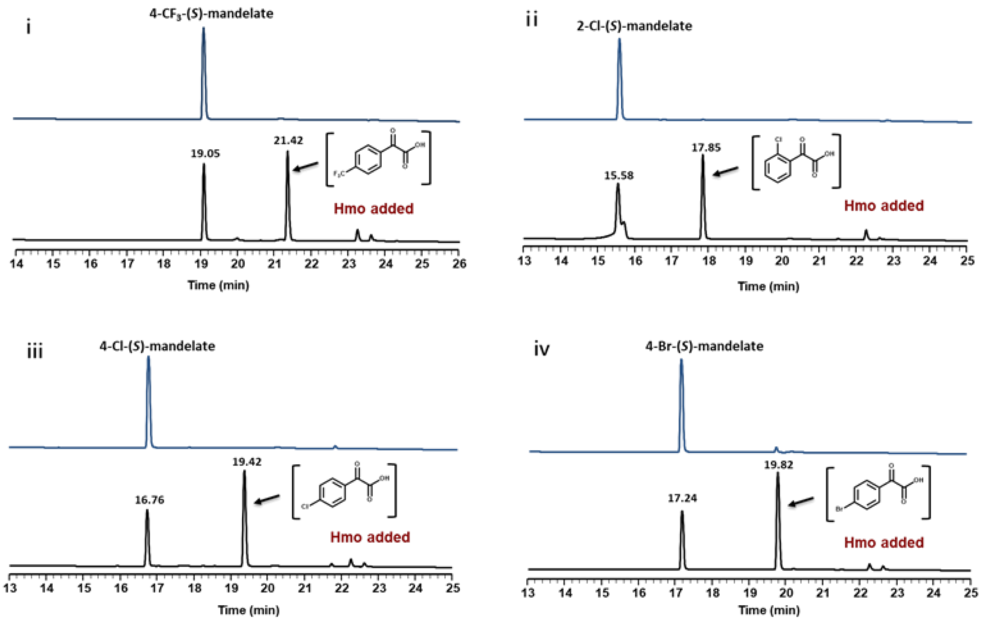
i
4-CF3-(S)-mandelate
19.05
21.42
Hmo added
Time (min)
ii
2-Cl-(S)-mandelate
15.58
17.85
Hmo added
Time (min)
iii
4-Cl-(S)-mandelate
16.76
19.42
Hmo added
Time (min)
iv
4-Br-(S)-mandelate
17.24
19.82
Hmo added
Time (min)

C

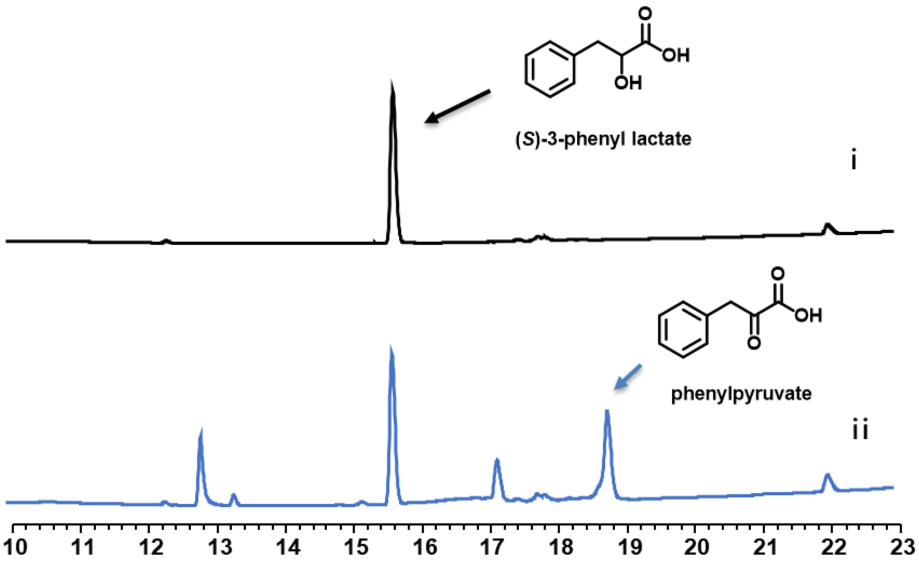

Figure S4 LC traces of mandelate analogs oxidized by Hmo.

(A) Enantioselectivity of Hmo: (*R*)-mandelate (i) cannot be oxidized by Hmo (ii), while (*S*)-mandelate (iii) can be oxidized by Hmo to benzoylformate (iv). (B) (*S*)-4-CF_3-mandelic acid (i), (*S*)-2-Cl-mandelate (ii), (*S*)-4-Cl-mandelate (iii), and (*S*)-4-Br-mandelate (iv) all can be oxidized to corresponding benzoylformate. (C) (*S*)-3-phenyllactate also can be oxidized to phenylpyruvate by Hmo.

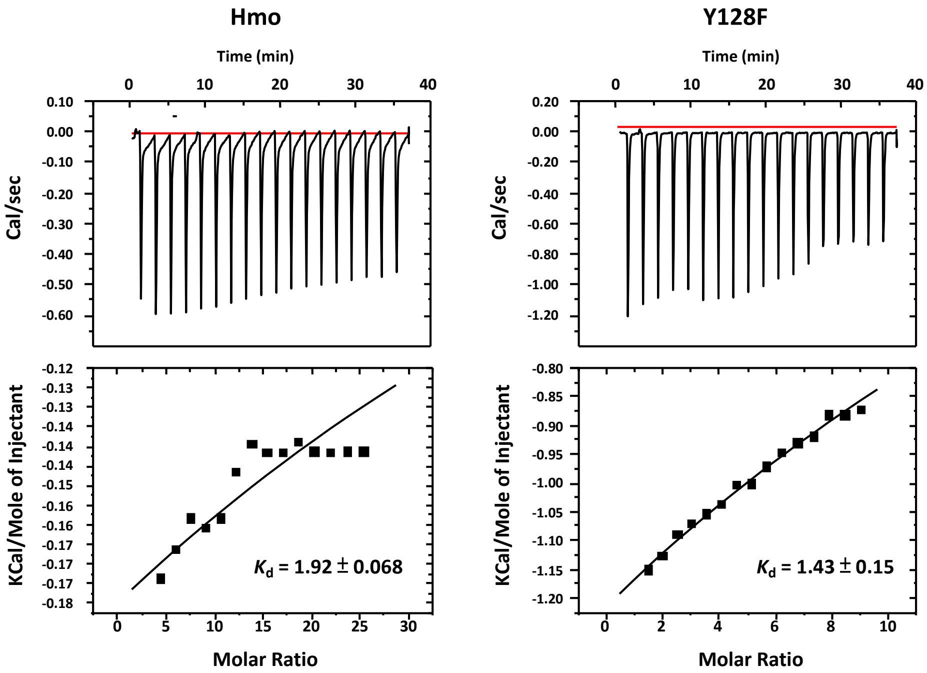

Figure S5 The binding affinity of benzoylformate versus Hmo or Y128F. The binding affinity of benzoylformate versus Hmo or Y128F was measured by isothermal titration calorimetry (ITC; MicroCal iTC200). The binding constants were estimated as K_d = 1.9 and 1.4 mM for the paris benzoylformate/Hmo and benzoylformate/Y128F, respectively, with the latter slightly better than the former. The binding affinity is weak by 1-2 orders of magnitude less than that of the substrate mandelate (0.1 ~ 0.07 mM).

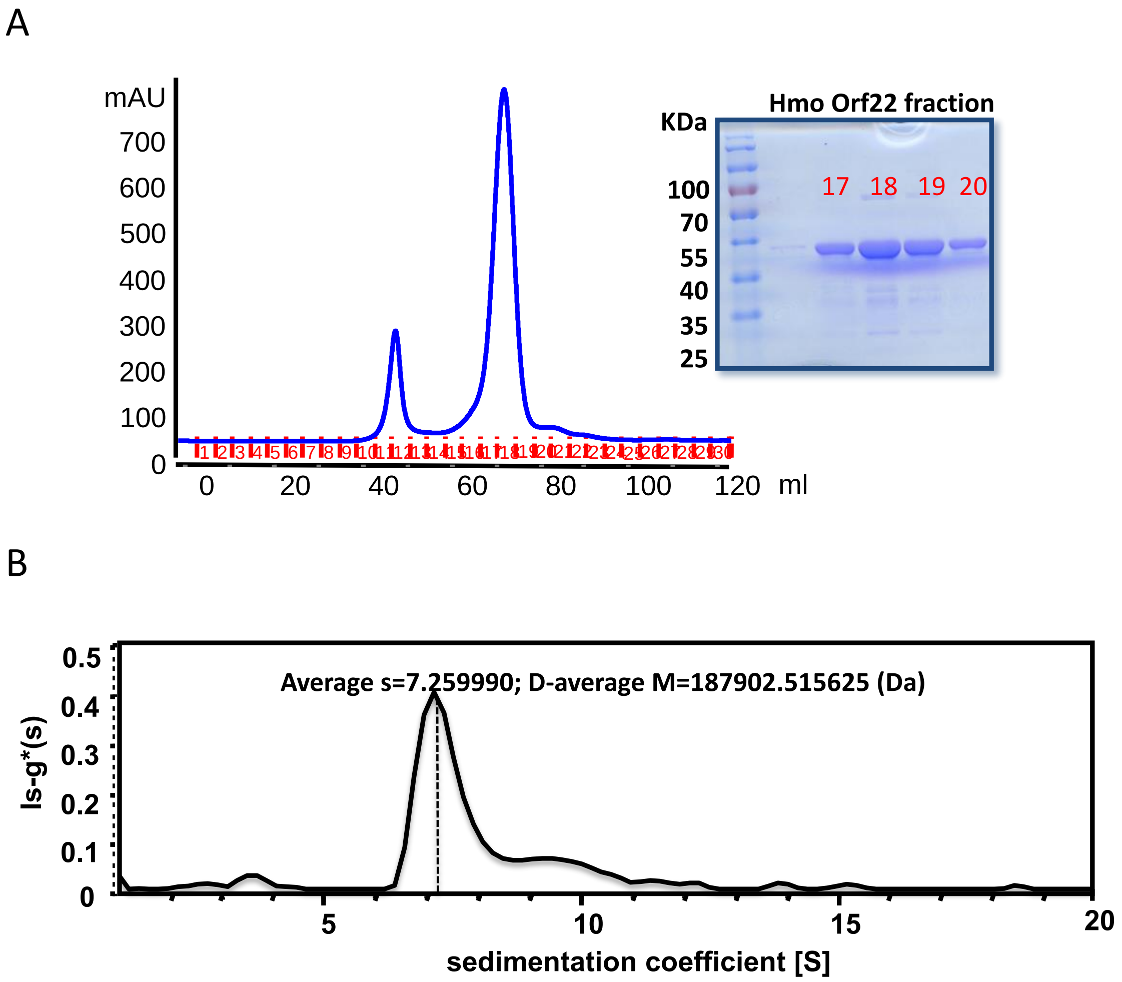

Figure S6 SDS-PAGE analysis, gel filtration chromatography and AUC of Hmo. (A) The spectrum of Hmo by fast performance liquid chromatography (FPLC) and the fractions at 17-20 tubes were analyzed by SDS-PAGE (inserted figure). (B) The sedimentation profile of Hmo determined by AUC with a molecular weight 187.9 g mol^{-1} equivalent to a tetramer (a dimer of dimers).

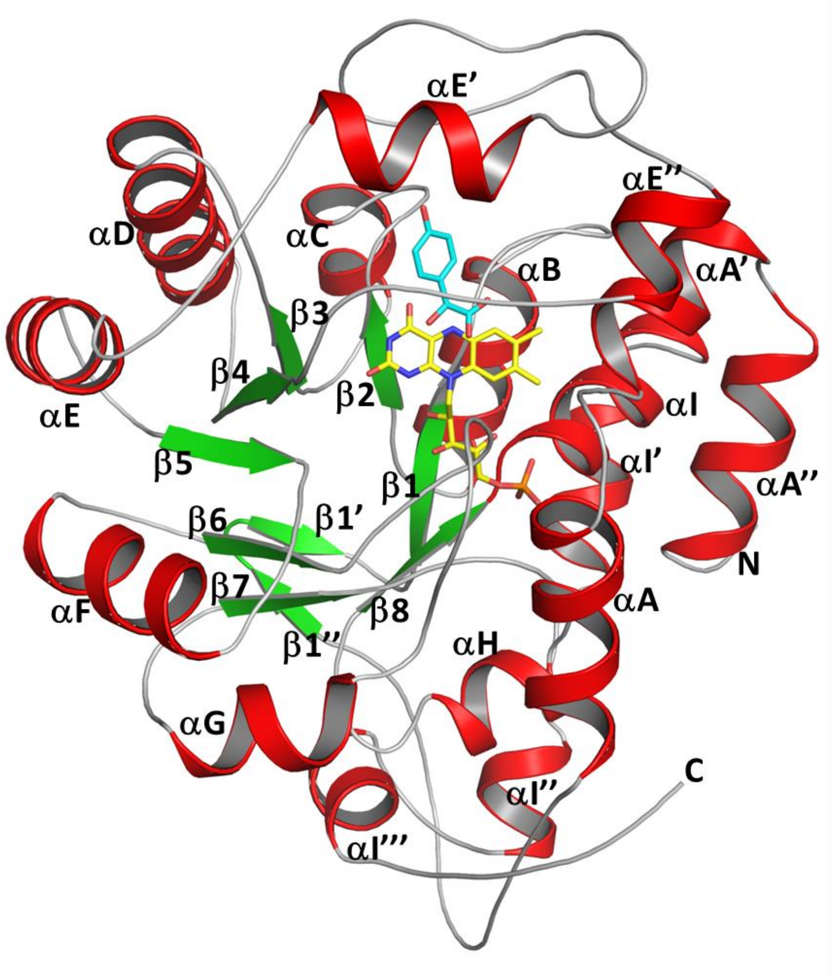

Figure S7 Overall structure of Hmo. Hmo is made of a single $(\beta/\alpha)_8$-barrel domain, known as TIM-barrel, where the C-terminus loops of β-strands of the barrel constitute the substrate and cofactor binding sites. One tightly-bound FMN cofactor is deeply buried with its redox-active isoalloxazine accessible to bulk solvents or substrates.

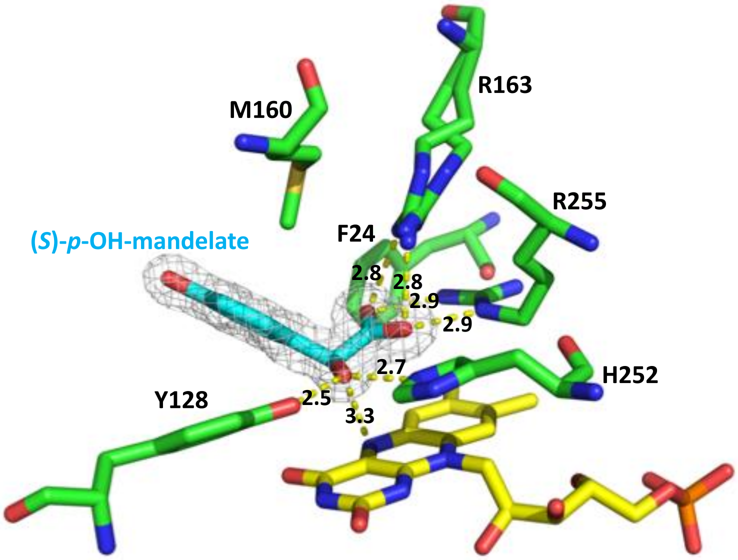

Figure S8 The crystal structure of Hmo in complex with (*S*)-*p*-OH-mandelate. The $2F_o$-F_c electron density map is contoured at 1 σ. The active-site residues, cofactor FMN and substrate 4-hydroxymandelate are colored green, yellow and cyan, respectively, in stick representation. The H-bond interactions in dashed lines and bond lengths in Å are indicated.

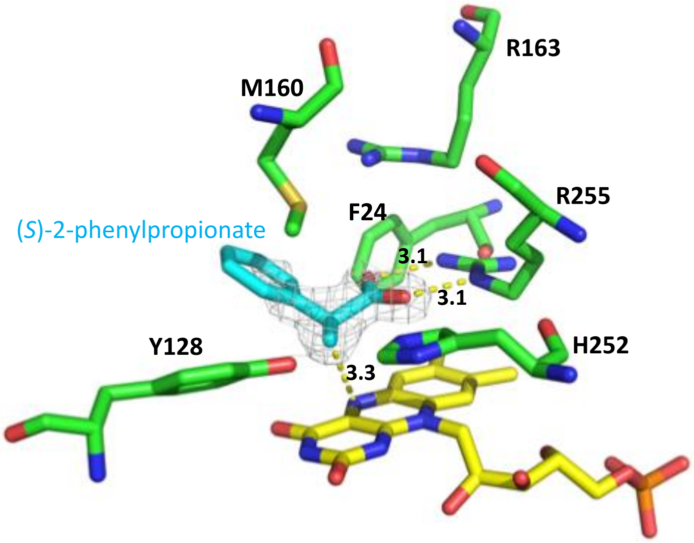

Figure S9 The crystal structure of Hmo in complex with (*S*)-2-phenylpropionate. The $2F_o$-F_c electron density map is contoured at 1 σ. The active-site residues, cofactor FMN and substrate (*S*)-2-phenylpropionate are colored green, yellow and cyan, respectively, in stick representation. The H-bond interactions in dashed lines and bond lengths in Å are indicated.

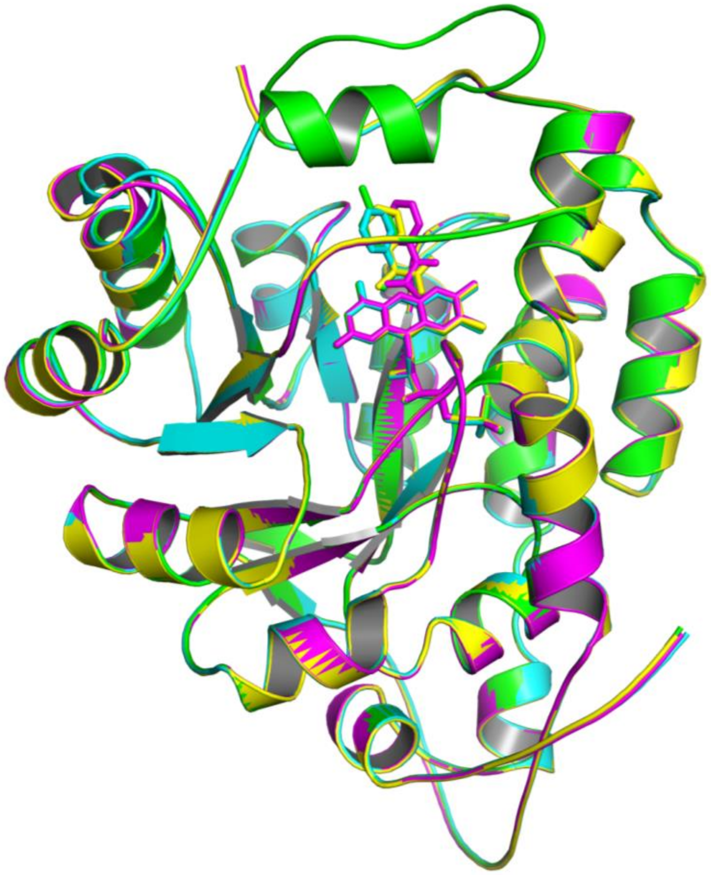

Figure S10 Superposition of ternary Hmo complexes. Superposition of the ternary complexes containing (*S*)-mandelate, (*S*)-*p*-OH-mandelate, benzoylformate or (*S*)-2-phenylpropionate shows low average root-mean-square deviations (rmsd) with 0.08, 0.12, and 0.12 Å, respectively, for Cα backbone atoms. Hmo in complex with (*S*)- mandelate, (*S*)-*p*-OH-mandelate, benzoylformate, or (*S*)-2-phenylpropionate is colored cyan, green, magentas or yellow, respectively.

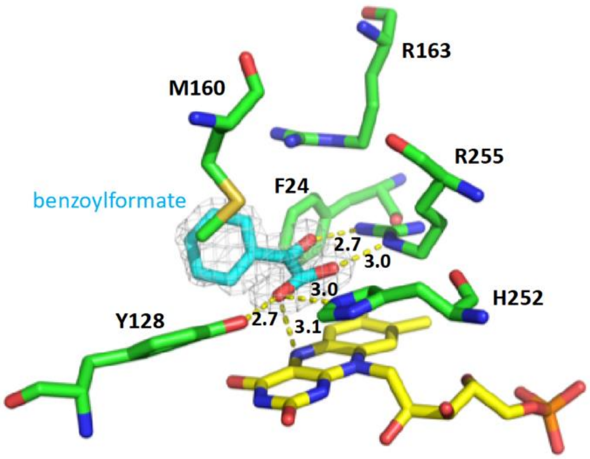

Figure S11 The crystal structure of Hmo in complex with benzoylformate. It almost always adapts to the *pro-R* orientation when crystals were soaked with benzoylformate. The $2F_o$-F_c electron density map is contoured at 1 σ. The active-site residues, cofactor FMN and substrate benzoylformate are colored green, yellow and cyan, respectively, in stick representation. The H-bond interactions in dashed lines and bond lengths in Å are indicated.

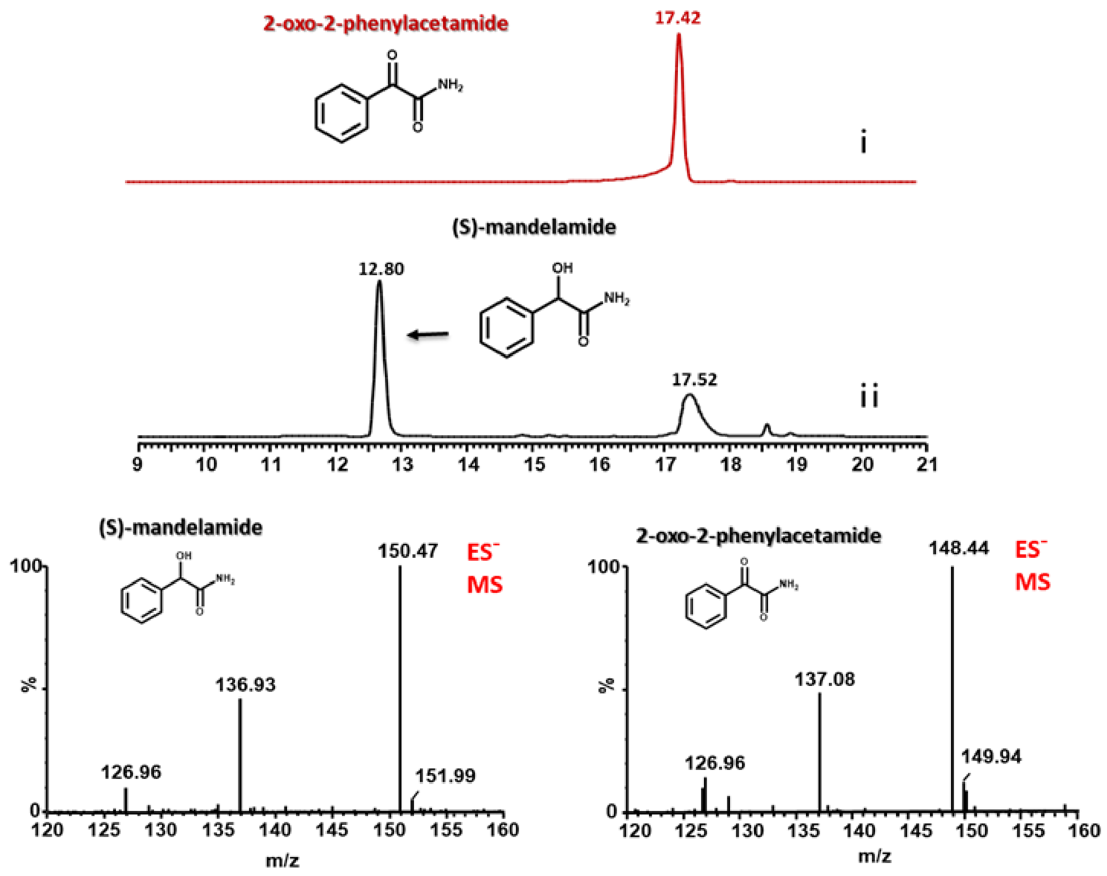

Figure S12 LC and MS spectra of mandelamide catalyzed by Hmo. (A) The LC traces of enzymatic reactions catalyzed by Hmo, where the substrate mandelamide in trace (i) is oxidized to phenylglycolic amide that is eluted with the same retention time as the synthetic authentic standard in trace (ii). (B) MS spectra of mandelamide (i, left panel) and phenylglycolic amide (ii, right panel).

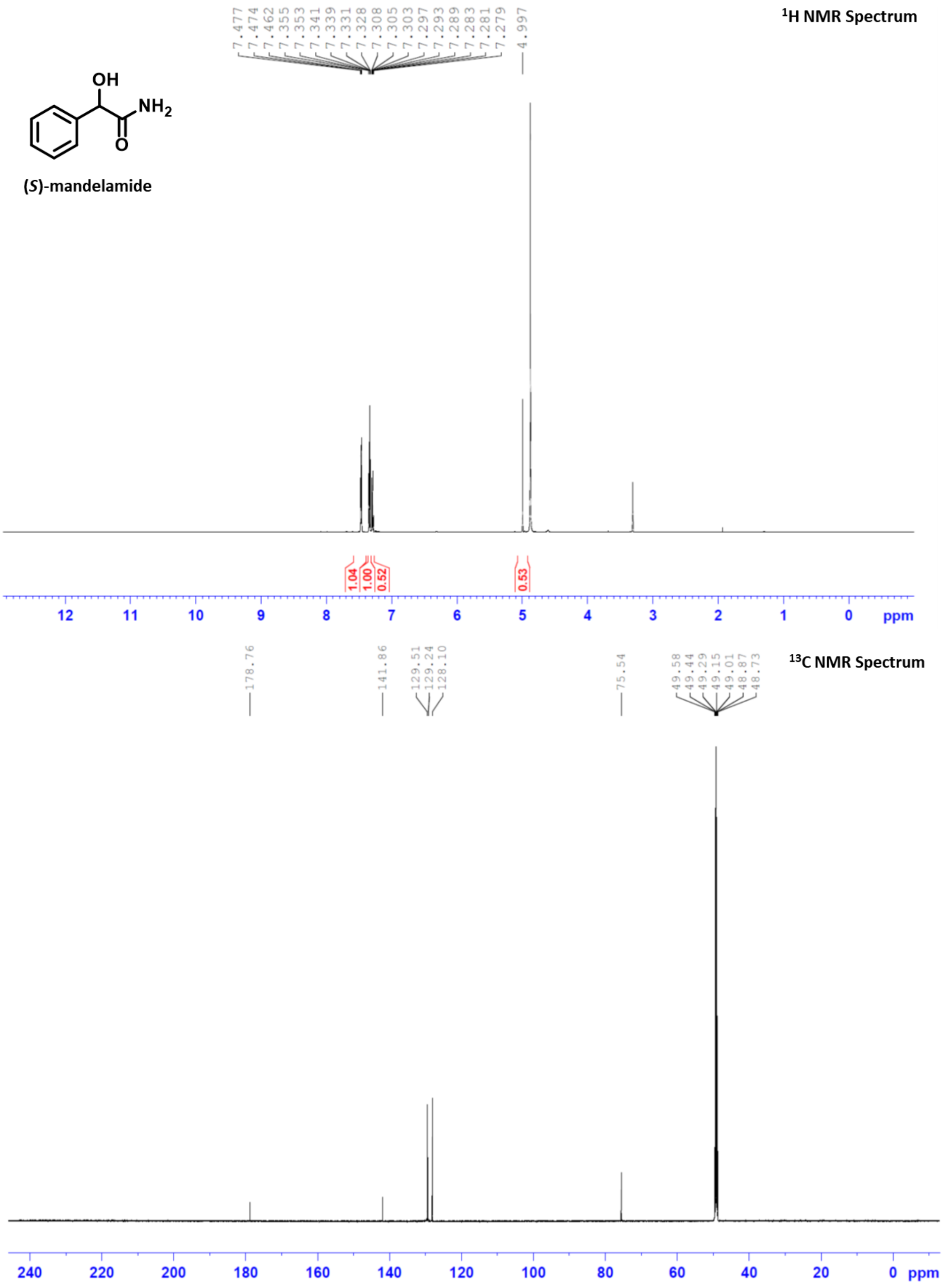

Figure S13 NMR analyses for (*S*)-mandelamide. NMR spectra include ^{1}H (top panel), and ^{13}C (bottom panel).

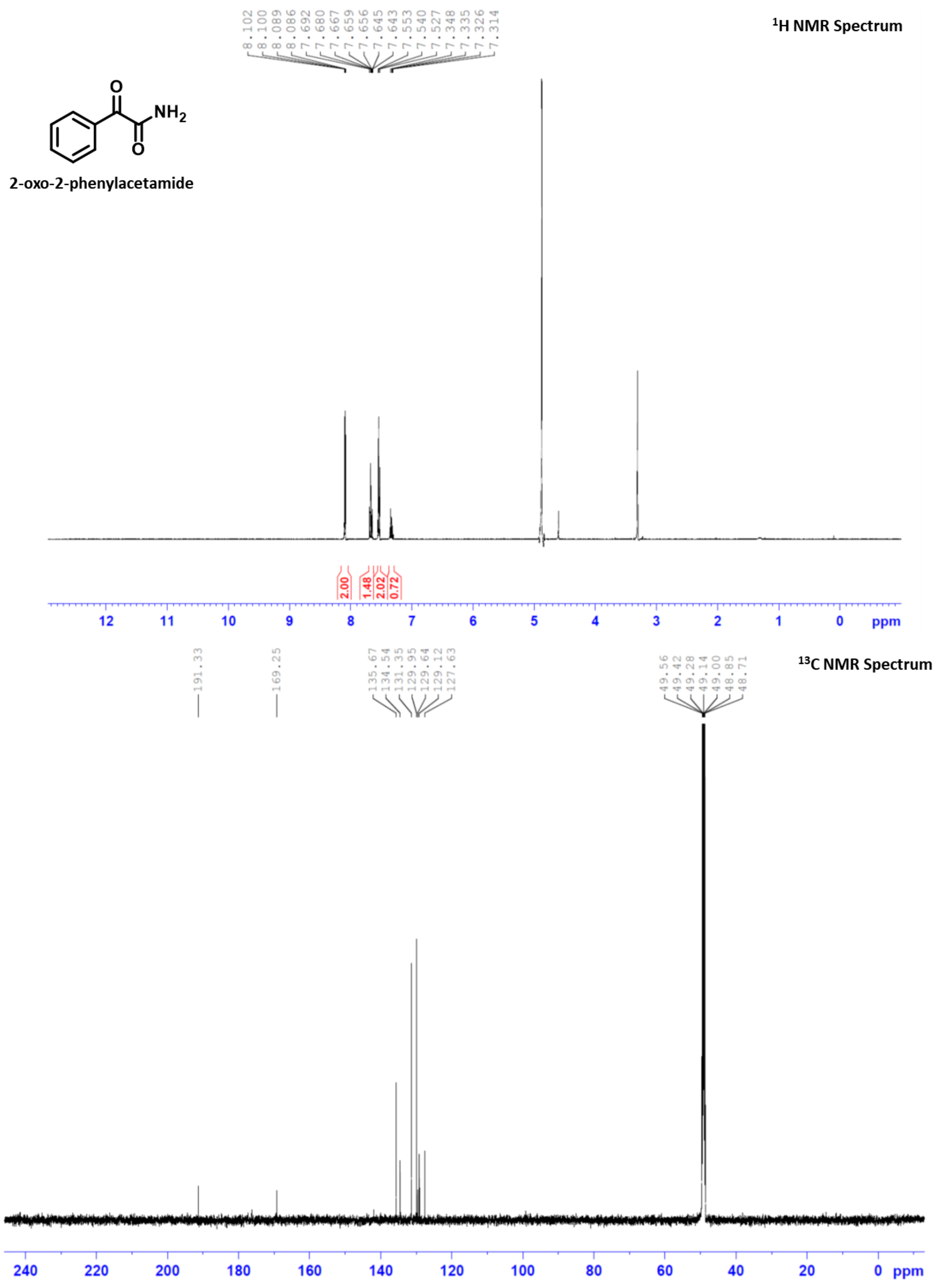

Figure S14 NMR analyses for 2-oxo-2-phenylacetamide. NMR spectra include ^{1}H (top panel), and ^{13}C (bottom panel).

Table S1 Primers used for preparing Hmo mutants

Y128A	
Forward	5'-CATCCCGCAGCCAGGCCAGCTGGAACCACG-3'
Reverse	5'-CGTGGTTCCAGCTGGCCTGGCTGCGGGATG-3'
Y128C	
Forward	5'-CATCCCGCAGCCAGCACAGCTGGAACCAC-3'
Reverse	5'-GTGGTTCCAGCTGTGCTGGCTGCGGGATG-3'
Y128F	
Forward	5'-CCGTGGTTCCAGCTGTTCTGGCTGCGGG ATGA-3'
Reverse	5'-TCATCCCGCAGCCAGAACAGCTGGAACCACGG-3'
R163L	
Forward	5'-CGTGGATGGGCAGGCTGTTGCGGGACATGCGC-3'
Reverse	5'-GCGCATGTCCCGCAACAGCCTGCCCATCCACG-3'
H252F	
Forward	5'-CCAACTGACGACCTCCGAAGTTGGACACCACGATTC-3'
Reverse	5'-GAATCGTGGTGTCCAACTTCGGAGGTCGTCAGTTGG-3'
R255A	
Forward	5'-AACCACGGAGGTGCCCAGTTGGACGGTGCC-3'
Reverse	5'-GGCACCGTCCAACTGGGCACCTCCGTGGTT-3'

Table S2 Kinetic analysis of Hmo/Y128F with various *p*-substituted mandelate substrates.

Substrates preferences of Hmo and Y128F mutant

**Hmo**	K_m (mM)	k_{cat} (1/s)	k_{cat}/K_m (1/M*s)
(*S*)-Mandelic Acid	0.1561	5.171	3.32×10^4
4-OH Mandelic Acid	0.0775	6.373	8.22×10^4
4-Br Mandelic Acid	0.3238	3.305	1.02×10^4
4-Cl Mandelic Acid	0.3591	6.634	1.85×10^4
2-Cl Mandelic Acid	0.1155	0.307	2.66×10^3
4-CF_3 Mandelic Acid	0.957	0.130	1.36×10^2

**Y128F**	K_m (mM)	k_{cat} (1/s)	k_{cat}/K_m (1/M*s)
(*S*)-Mandelic Acid	0.132	0.056	4.24×10^2
4-OH Mandelic Acid	0.1886	0.155	8.22×10^2
4-Br Mandelic Acid	0.4067	0.029	0.71×10^2
4-Cl Mandelic Acid	0.4625	0.052	1.12×10^2
2-Cl Mandelic Acid		N.D.	
4-CF_3 Mandelic Acid		N.D.	
